# An Integrated Approach Is Needed for Ecosystem Based Fisheries Management: Insights from Ecosystem-Level Management Strategy Evaluation

**DOI:** 10.1371/journal.pone.0084242

**Published:** 2014-01-13

**Authors:** Elizabeth A. Fulton, Anthony D. M. Smith, David C. Smith, Penelope Johnson

**Affiliations:** 1 Wealth from Oceans Flagship, Commonwealth Scientific and Industrial Research Organisation, Hobart, Tasmania, Australia; 2 Rural Environment and Agriculture Statistics Branch, Australian Bureau of Statistics, Hobart, Tasmania, Australia; Aristotle University of Thessaloniki, Greece

## Abstract

An ecosystem approach is widely seen as a desirable goal for fisheries management but there is little consensus on what strategies or measures are needed to achieve it. Management strategy evaluation (MSE) is a tool that has been widely used to develop and test single species fisheries management strategies and is now being extended to support ecosystem based fisheries management (EBFM). We describe the application of MSE to investigate alternative strategies for achieving EBFM goals for a complex multispecies fishery in southeastern Australia. The study was undertaken as part of a stakeholder driven process to review and improve the ecological, economic and social performance of the fishery. An integrated management strategy, involving combinations of measures including quotas, gear controls and spatial management, performed best against a wide range of objectives and this strategy was subsequently adopted in the fishery, leading to marked improvements in performance. Although particular to one fishery, the conclusion that an integrated package of measures outperforms single focus measures we argue is likely to apply widely in fisheries that aim to achieve EBFM goals.

## Introduction

Globally, changes in the focus of public and scientific scrutiny has seen the concept of sustainable fisheries evolve to incorporate concern for the wider ecological impacts of fisheries on marine ecosystems [1]–[5]. Consumers and policy makers are now paying much closer attention to ecological impacts of fishing (expanding beyond target species to non-target species, food webs and habitats), leading to a keen interest in the most effective means of executing ecosystem based fisheries management (EBFM) and ecosystem based management (EBM) more broadly (the latter considering all uses and stressors of marine and coastal systems). Unfortunately, there has been a tendency to seek simple and straightforward solutions to the fishery management “problem” with a desire to identify the “silver bullet” solution that will solve all management issues [6]–[7]. The form that EBFM should take remains a contentious topic among the various disciplines interested in fisheries management: ecology, fisheries science, economics and social science. Across different groups and at various times the following forms of management have all had vocal advocates: strong top-down regulation (e.g. using gear, effort, catch or seasonal controls [8]); incentive-based approaches and strong property rights [9]–[12]; community-based management [10]–[13]; portfolio-based management [13]; and spatial management [14]–[17]. Reviews of the performance of each of these approaches suggest that no single solution will perform well in all circumstances and for all species [6], [18]–[19] leading to the view that the various options should be considered as parts of a larger tool kit rather than as stand-alone measures. However, the tone of the debate in the literature has often been adversarial leaving fisheries managers seeking guidance on what measures or combination of measures will best meet the multiple and often conflicting objectives of EBFM.

Management strategy evaluation (MSE) is a decision support tool that has seen wide use in fisheries management [20]. It is a simulation-based approach that is used to explore alternative management options and to identify the trade-offs across a range of management objectives. Its main application has been in exploring single species harvest strategies, but the method has also started to be applied to broader aspects of fishery management, including EBFM [21]. In this paper we describe the application of the MSE approach to a complex multispecies fishery and use the results to draw more general conclusions about the range of measures that are likely to feature in practical implementation of EBFM.

### A complex multispecies fishery

**Figure 1:**
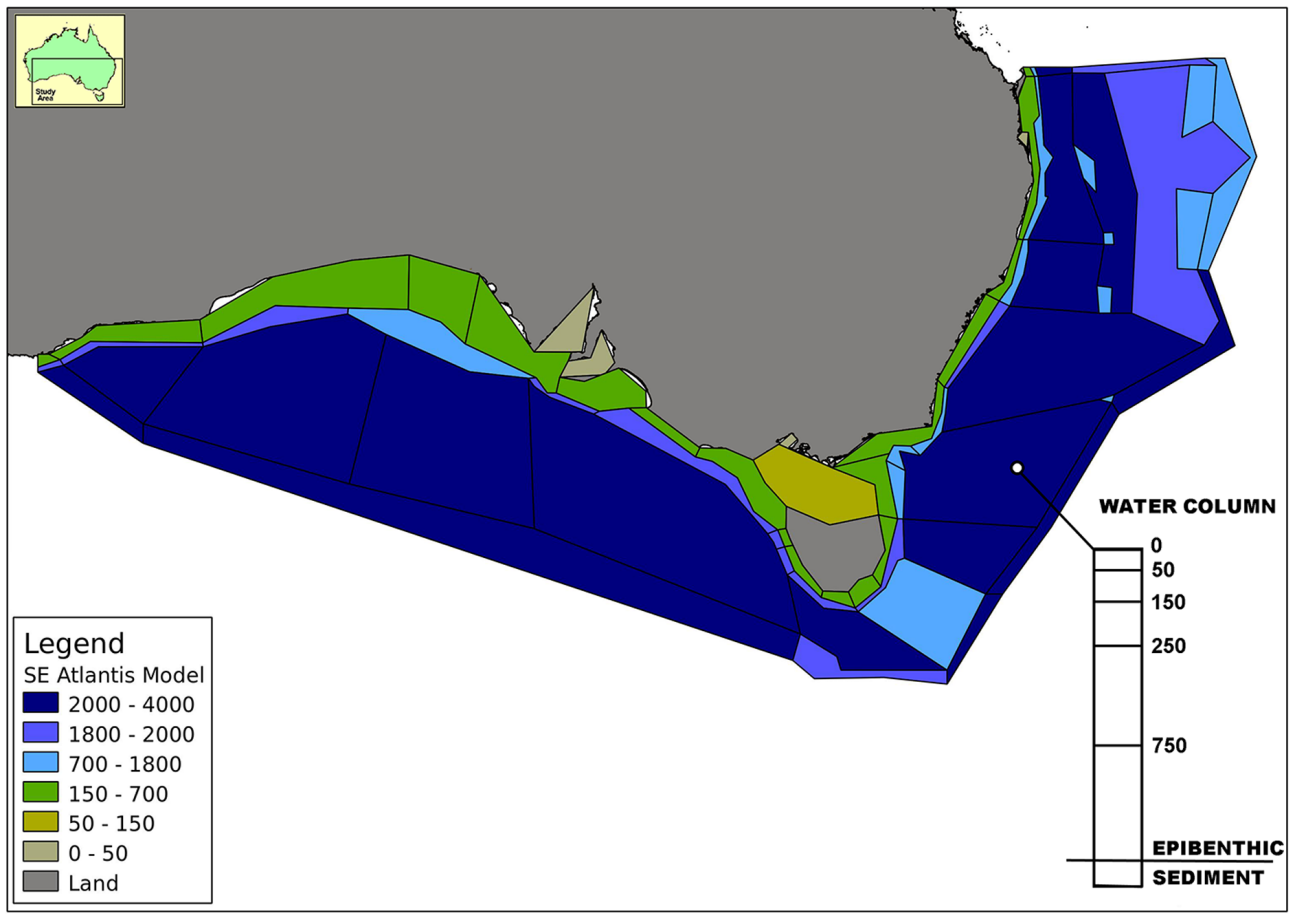
Map of the southeast region, for reference model geometry is shown in light grey.

Australia's Southern and Eastern Scalefish and Shark Fishery (SESSF) spans 3 million km^2^ (Figure 1) from sub-tropical to cool-temperate latitudes and involves multiple fishing methods and fleets targeting over 30 commercial species with hundreds of other species caught incidentally [22]. The SESSF is a cost-recovery fishery managed by the Australian Fisheries Management Authority (AFMA) and is subject to both fishery and environmental legislation. The list of management objectives for the fishery is long (Table 1).

**Table 1:** List of key objectives for the defined by Australian Fisheries Management Authority (AFMA) legislation, the Environment Protection Biodiversity Conservation (EPBC) Act 1999 and Australia's National Plan of Action for the Conservation and Management of Sharks.

Type of objective	Specified objective
Overall	• the integration of both long-term and short-term economic, environmental, social and equity considerations
	• adherence to the precautionary principle
	• the implementation of harvesting strategies consistent with the principles of biological sustainability and rational long-term economic use
	• facilitation of ecological monitoring, catch and economic reporting
	• accountable management executed under a cost recovery structure
Ecological	• the conservation of biological diversity and ecological integrity
	• prevention of activities that could cause significant impact to a listed threatened, endangered or protected species or habitat (including all marine environments)
	• minimisation of waste and discards
Economic	• improved valuation, pricing and incentive mechanisms to be promoted
	• efficient and cost-effective fisheries management; maximisation of net economic returns
Social	• inter-generational equity
	• promotion of a co-operative approach to the protection and management of the environment involving governments, the community, land-holders (or rights-holders) and indigenous peoples

The SESSF has a complex history in terms of the form of fishing activities, shifting management focus and the interplay of multiple drivers. Fishing commenced in the first decades of the 20^th^ century and fishing methods have included trawl, Danish seine, gillnets, line fishing and traps. The fishery was managed using input controls up to the mid 1980s, but these measures were gradually replaced with a quota management system on over 20 species and individual transferable quotas (ITQs) were implemented in 1992. However by the late 1990s and early 2000s the SESSF faced severe problems, including poor economic performance of several key sectors and a deteriorating ecological status, including a number of overfished species [23]. There was general agreement among stakeholders (including managers, industry, scientists and environmentally focused non-government organisations (eNGOs)) that the management measures in place in 2004 were failing to address the ecological and economic goals laid out under Australian legislation and were not meeting social needs and expectations. This led to broad stakeholder acknowledgement that it was time to rethink management directions and resulted in a formal study to evaluate alternative management options.

This paper reports on the evaluation of four of the strategies that were explored: (i) 2003 status quo; (ii) enhanced quota management; (iii) integrated combination of management measures (including quota management, effort management, gear controls, and spatial management); and (iv) conservation dominated management. A broader set of potential strategies (with more subtle differentiation between strategies) was considered by [24]. This body of work is too large to report here and readers interested in the entire set of management strategies and background scenarios (e.g. for human population or environmental drivers) are directed to that technical report. However, the strategies presented here cover the range of alternatives evaluated in the broader study.

## Methods

The stakeholder process for the SESSF required the evaluation of a range of alternative management strategies, where each strategy comprised a range of management measures. The stakeholders had also defined a range of ecological, economic and social objectives to be met. Given these requirements, the problem seemed best suited to a Management Strategy Evaluation (MSE) approach. The key steps in an MSE study include [20]:

1. Specifying management objectives
2. Developing performance measures for each objective
3. Identifying a range of management strategies (and contextual scenarios)
4. Predicting the consequences of applying each management strategy
5. Evaluating trade-offs and communicating with decision makers

The Atlantis modelling framework was used to perform the MSE. The Atlantis framework uses a deterministic biogeochemical whole of ecosystem (or end-to-end) model that includes modules for each of the major steps in the adaptive management cycle (making it well suited for use in MSE [21]). A full description of the Atlantis framework and the various stages of its implementation, calibration and strategy evaluation is beyond the scope of a single paper. This paper is restricted to consideration of the MSE undertaken using the Atlantis framework (this implementation of the Atlantis framework is known as Atlantis-SE), with only a brief overview of the model details and historical fit to data provided in Text S1 (including lists of the biological groups and fleets models – Tables S1 and S2 in Text S1). A detailed description of the underlying Atlantis modelling framework is given in [24]–[25]; and a full description of the parameterisation of this model is given in [24].

For the purposes of evaluating the modelled management strategies the key points about Atlantis to note are: that it is a 3D mechanistic whole of system model tracking the key biophysical components of the physical environment and (age-resolved) food webs in the region on a diurnal time step; that the main fleets (or metiers) are tracked at the same spatiotemporal resolution; that effort allocation by the fleets includes social, psychological and economic drivers; and that snapshots of the entire system are stored quarterly with standard system-level biomass and economic indices reported annually. Consequently the various performance measures (detailed further below) can be compared between simulations to determine the relative ranking of the management strategies against the suite of objectives that have been defined for the SESSF. For the purposes of this exercise the projection period extended 20–40 years after an initial 10 year burn-in period and then a 10 year period replicating historical management and fishing conditions. The 10 year burn-in period has previously been shown to be sufficient as the starting conditions (including the age structure of populations etc.) are taken from the end point of a century-long calibration simulations fitted to historical time series – see Text S1, Figure S2 and Figure S3 for further details. To deal with uncertainty the simulations were conducted using an ensemble of parameterisations and model structural configurations (see Text S1 for further details).

### Management strategy descriptions

The Atlantis modelling framework can mimic the implementation of all major fisheries management options, allowing for consideration of quite sophisticated (and complicated) management options. The alternative management strategies trialled in the model and reported here were developed with the assistance of, and approved by, a stakeholder steering committee. Membership included fishery and conservation managers, industry representatives and eNGOs.

The four strategies are briefly outlined below. Table 2 contains further details of each strategy, including the quota, spatial management, input, gear and other controls in place in each case. A map of the spatial management options used is given in Figure S1.

**Table 2:** Summary of management strategies used in management strategies explored with Atlantis-SE.

Management Control	Strategy 1	Strategy 2	Strategy 3	Strategy 4^1^
*Logic*	2003 Status quo	Enhanced quota management	Integrated management	Conservation dominated management
*Total Allowable Catch (TAC)*				
Method for setting TAC	Catch trends-based	Based on dynamic assessments	Based on dynamic assessments	Based on dynamic assessments
Frequency of assessment	Annual	1–3 years	1–3 years	1–3 years
Number of species (groups) under TAC	17 target species	30 target, bycatch & conservation species	17 target species + gulper sharks	17 target species
Tiered harvest control rules used	No	Yes	Yes	Yes
Non-quota species under TAC	No	Yes (Baskets^2^)	Yes (Baskets^2^)	No
Companion TACs	No	Weak stock limiting	Weak stock limiting	No
Accounting for discards (against quota)	No	No	Yes	Yes
Regional TACs	No	Yes	Yes	Yes
Quota reconciliation	Annual	Before landing	Before landing	Before landing
*Spatial management (also see Figure S1)*				
Fisheries zoning	Yes (largely ineffective)	Yes	Extensive (differential access by gear type)	Very extensive (up to 80+% closure of some habitat types)
Fishery closures (no take)	No	No	Yes and voluntary spawning closures	Yes and compulsory spawning closures
Sectoral closures (by method)	Those existing in 2003	Additional (based on depth and gear)	Extensive closures outside well established existing grounds.	Extensive across the board.
Industry closures	No	No	No	No
Compliance with zoning	Variable^3^	Variable^3^	Variable^3^	High
*Gear controls*				
Trawl – mesh size	90 mm	90 mm	100 mm	110 mm
Prawn trawl – mesh size	40–60 mm	40–60 mm	40–60 mm	40–60 mm
Danish seine – mesh size	38 mm	38 mm	38 mm	45 mm
Gillnet – length, height, mesh size	150–165 mm, 5000 m set	150–165 mm, 5000 m set	150–165 mm, 5000 m set	150–165 mm, 6000 m set
Auto longline – no. hooks/licence	15,000 hooks per set	15,000 hooks per set	15,000 hooks per set	Unlimited
Drop line, trap, shark long line	As of 2003	As of 2003	As of 2003	As of 2003
Restrictions on new fishing methods	No	Yes	Yes	Yes
Bycatch Reduction Devices	Poor uptake of voluntary reduction of bycatch	1–80% reduction in bycatch rates	Compulsory bycatch reduction (by 1–80%)	Compulsory bycatch reduction (by 1–80%)
Targeting	Unchanged	50–80% reduction in discarding rates		
*Input controls*				
Limited entry	Yes	Yes	Yes	Yes
Increase in number of licences^4^	No	No	No	No
Buy back of licences	No	No	No	Yes (after 5 years)
Choice of gear	No	No	Yes (can switch gear or vessel size)	Yes
Vessel length (GABTF)	Yes	No	No	Yes (for auto longliners)
*Other*				
Logbooks and landing records	Yes	Yes	Yes	Yes
Observer coverage	25%^5^	25%^5^	25%^5^	25%^5^
Fishery independent survey	No	Yes (largely acoustic)	No	Yes (largely acoustic)
Discard monitoring and enforcement	No	No	Yes	No

Evolving indicates the gear control is changing through time in the course of the simulation (through variable uptake or staged implementation), while increasing indicates a relaxation (an increase) in the number of hooks allowed for use by auto longline.

^1^ . Drawn up by the Australian Marine Conservation Society to emphasise conservation and recovery of overfished and threatened marine species by mitigating fishing impacts on species, food webs and habitats and enhancing productivity in fished areas.

^2^ . Basket quotas: where a species group (e.g. all Oreos, or all sharks taken from the upper slope) are under a single cumulative quota.

^3^ . Without increases in enforcement, compliance remains a dynamic decisions made by simulated fishers and so remains variable across personality types, vessel classes and sectors based on the changing conditions through time.

^4^ . Licensees may retire licences (or let them sit latent) if they can't profitably execute them, though they will attempt to sell the licence if their criteria for sale are met.

^5^ . Not fisheries independent, it is only recording information on catch and discards, but it can influence the error rates assumed by the assessment method and rates of compliance.

#### 2003 Status quo

No change to the 2003 status quo management arrangements, with the exception of the assumption of a voluntary uptake of larger mesh sizes in the trawl industry and a small expansion of spatial management to provide some measure of habitat protection. The fishery is a limited entry licence-based fishery with individual transferable quotas applied to 17 key target species; the level of the annual quota is dictated by a total allowable catch (TAC) estimate calculated from catch trends. Vessels may fish without quota on the proviso that all landings are reconciled with quota (owned or leased in) by the end of the calendar year. A small proportion of the EEZ is closed to specific fishing gears (and enforcement is low). Gear size and power restrictions (of mixed effectiveness) exist for trawl gear, longlines, drop lines and traps. Catch reporting is in the form of compulsory logbooks and monitoring is fishery dependent (25% observer coverage of fishing vessels).

#### Enhanced quota management

Major changes to the quota management system (QMS) in relation to the 2003 status quo strategy, but most input controls are retained. This strategy envisages the ongoing extension of the QMS as the main tool applied to future management of the fishery. Under this strategy, all species of commercial or conservation concern (beginning with the 30 most important commercial, bycatch and endangered species) come under the QMS through application of single species or basket quotas (where quota is set in aggregate for a group of species rather than individually). These quota are set on a 1–3 year cycle (key target species annually, data poor bycatch and endangered species every 2–3 years), follow tiered harvest control rules [26] and are resolved to regions thought to match genetic stocks or bioregions (see Text S1 for further details). The use of multi-year and basket TACs is proposed as a means of providing a practical system for handling a large number of minor species within the quota system. Quota reconciliation occurs at the time of landing rather than at the scale of the calendar year. The strategy also involves the application of spatial controls for the major sectors, based on controlling the depths that specific gear types may access to minimise physical gear interactions and reduced tensions between sectors due to gear disturbance or destruction. Additional gear restrictions are also implemented – in particular, the introduction of bycatch reduction devices (with an up to 80% reduction in bycatch and discarding rates). Fishery dependent observing and reporting requirements are identical to the 2003 status quo arrangements, with the addition of a fisheries independent acoustic survey of key target species.

#### Integrated management

This strategy explores taking a balanced approach across all management options, tailoring their implementation to what will best address each of the fishery objectives in turn. In this strategy regional quotas are set for 17 key target groups and groups of conservation concern (e.g. gulper sharks) on a 1–3 year cycle, following tiered harvest control rules. Quota is required to be reconciled at the time of landing and discards are accounted for in the quota setting process. This strategy also sees wider use of spatial management to deal with objectives that cannot be addressed in other ways – including specifying separate areas of gear operation (to minimise conflict over gear interactions) and extensive use of area closures to protect key habitats, spawning locations of target or vulnerable species and the like. The other major management modifications are removal of restrictions on the fishing method that may be used by each sector and the monitoring and enforcement around discarding.

#### Conservation dominated management

Under this strategy, the quota management system remains in place for the 17 key target species. Regional quotas are set on a 1–3 year cycle, following tiered harvest control rules, with the requirement to account for discarding and the reconciliation of landings against quota at the time of landing. Stringent restrictions on vessel size and power are introduced to constrain effort, although gear restrictions (i.e. gear type to be used per sector) are relaxed; with effort intentionally reduced through a scheduled buy-back of licenses and retirement of associated boats 5 years after the management strategy begins.

The key difference between this strategy and the other three is the definition of a detailed and comprehensive system of spatial management zones. This inverts the typical marine zoning paradigm of “most areas open to exploitation, with a moderate proportion closed to exploitative behavior”. Instead this strategy closes large areas of the fishery (as much as 80% or more of some habitat types), leaving open clearly defined fishing “paddocks” (i.e. areas of fishable sea bottom open to fishing) in each depth zone outside of 3 nm and shallower than 800 m (all areas closer to shore than 3 nm or deeper than 800 m are closed to all SESSF fishing).

### Performance measures

A range of indices were used to calculate overall integrated performance measures for judging the trade-offs between objectives and the relative performance of the different management strategies (see Table 3). These indices matched all the major objectives defined for the fishery, covering: the biological status of target, bycatch and habitat forming species; biodiversity and ecosystem structural integrity; economic status of the fishery; operational indices for the industry (e.g. access to product); non-economic industry measures (e.g. catch and effort); and social considerations.

**Table 3:** List of indices used as performance measures when considering the performance of a management strategy versus objectives.

Class of performance measure	Index	Notes
Non-economic industry measures	Overall discards*	Summed over all species
	Habitat-impact*	Area impacted
	TEP interactions*	
	Total effort*	
	Total landings	
	Catch-per-unit-effort	Target species
	Average size (cm) of the catch	
	Catch composition*	Maximum proportion of catch due to one species
Operator perception measures	Access to fishery	Reflects spatial or other regulatory constraints
	Stability of management	Year-to-year
	Volume of quota trading	
Measure of management costs	Overall management costs*	General administration
	Research costs*	Ecological data for assessments
	Enforcement costs*	Patrolling; VMS costs
	Costs of monitoring*	Surveys & observers
	Assessment costs*	
Social measures	Public image	Based on the relative state of the ecological system, port population size and profitability of the system
	Frequency of gear conflict*	Count of adverse gear interactions
	Level of port activity	Function of landings
Economic measures	Gross value of landed catch	
	Total operating costs*	Summed over fuel, gear, refrigeration & transport, unloading, capital & fixed costs.
	Total profits	With a penalty function for losses
	Profit per tonne landed	Across all species
	Profit per effort	
Ecological measures	Biomass of target species	
	Biomass of bycatch species	
	Microfaunal biomass*	
	Biomass of threatened, endangered & protected species	
	Biomass of higher trophic level species	Sea birds, marine mammals, large sharks
	Proportional habitat cover	Per habitat type
	Demersal:pelagic biomass ratio	Of finfish
	Piscivore:planktivore biomass ratio	Of finfish
	Change in slope of biomass size spectra*	

When combined into composite “overall performance indices” those measures marked with a * were inverted, so that a high value always indicated a better performance with regard to management objectives.

As mentioned above, the performance of the strategies were assessed using 20–40 year projections under multiple parameterisations. When considering the performance of individual strategies these performance measures are considered individually, whereas for comparison across strategies they are pulled together into composite indices. For ease of interpretation and visualisation, the final composite values were then normalised over all strategies and all years so that the best result of a performance measure is assigned a value of 1 and all other values scaled accordingly.

## Results

**Figure 2:**
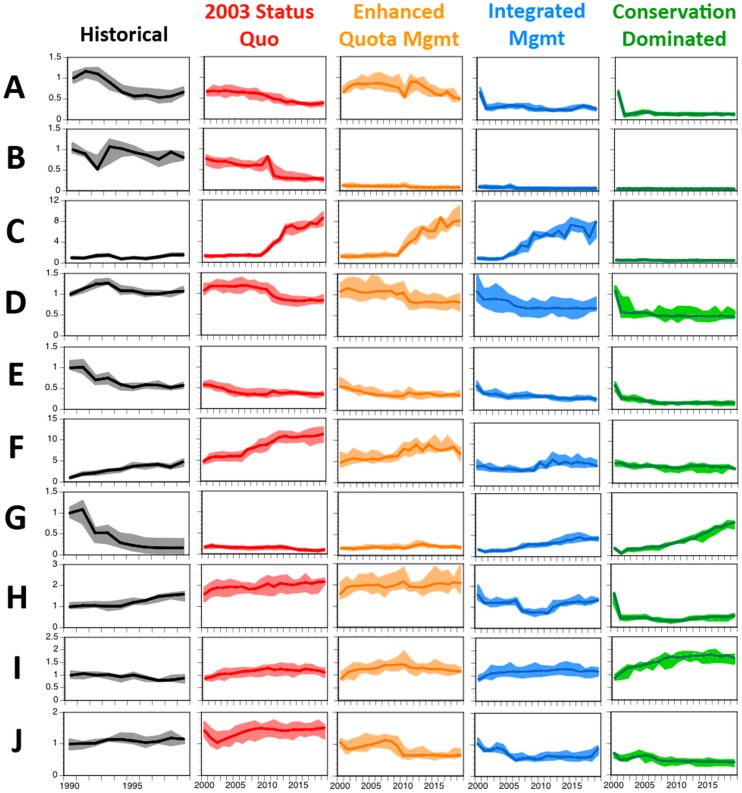
Trajectories for the non-economic industry measures through a historical period and under each management strategy. (A) overall discards, (B) habitat impact (C) interactions with threatened, endangered and protected species, (D) total effort, (E) total landings, (F) catch-per-unit-effort for low trophic level groups, (G) catch-per-unit-effort for high trophic level groups, (H) trip length, (I) average size of the catch and (J) catch composition. All are shown relative to the values in 1990. Dark line is trajectory from best-fit parameterisation with coloured areas showing lower and upper quartile of results across all parameterisations used.

**Figure 3:**
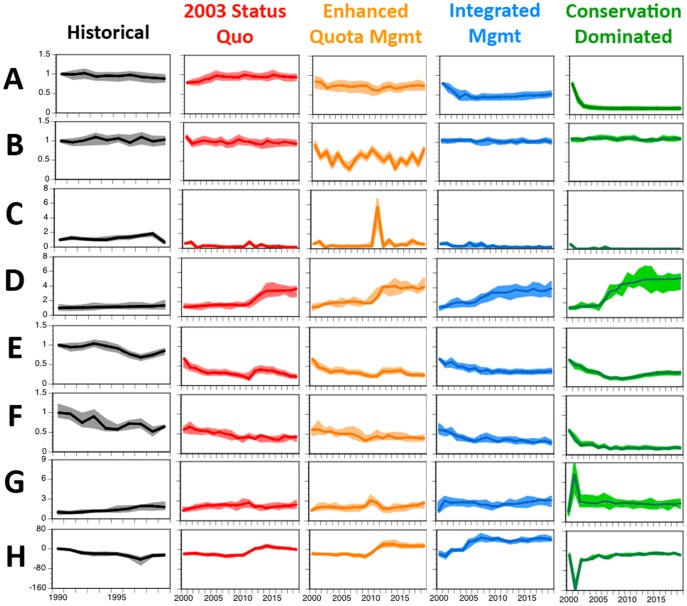
Trajectories for operator perception measures, social indices, management and other economic costs through a historical period and under each management strategy. (A) access to the fishery, (B) stability of management, (C) volume of quota trading, (D) management costs per boat, (E) public image, (F), level of port activity, (G) operating costs per tonne and (H) profit per tonne landed. All are shown relative to the values in 1990. Dark line is trajectory from best-fit parameterisation with coloured areas showing lower and upper quartile of results across all parameterisations used.

**Figure 4:**
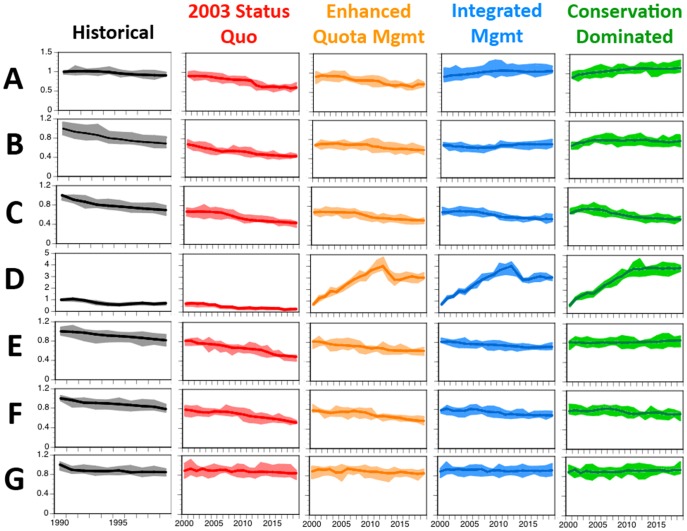
Trajectories of overall ecological indices through a historical period and under each management strategy. (A) diversity, (B) relative biomass of target species, (C) relative biomass of bycatch species, (D), relative biomass of higher trophic level species, (E) proportional habitat cover, (F) demersal:pelagic biomass ratio and (G) piscivore:planktivore biomass ratio. All are shown relative to the values in 1990. Dark line is trajectory from best-fit parameterisation with coloured areas showing lower and upper quartile of results across all parameterisations used.

**Figure 5:**
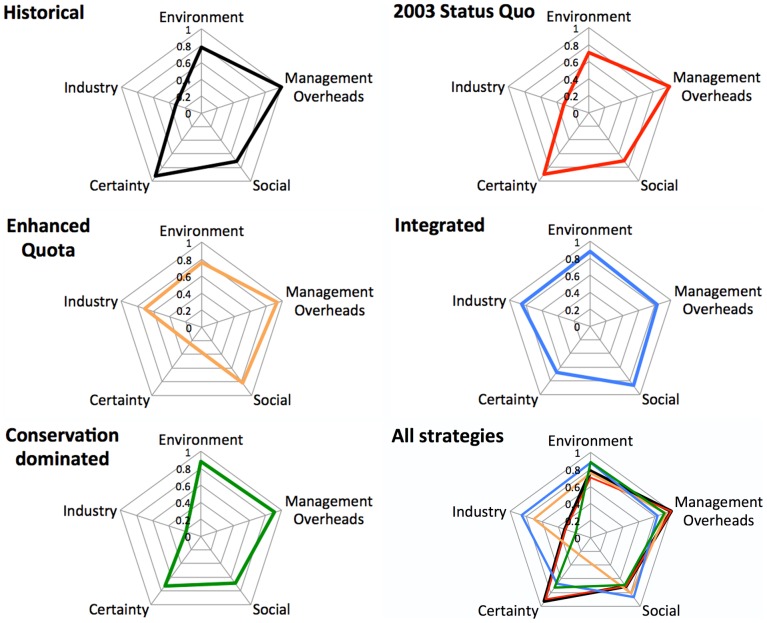
The overall performance of the management strategies for the scaled integrated (composite) performance measures (normalised so 1.0 = good and 0.0 = poor performance). The historical state and each strategy is shown separately and for comparative purposes all the strategies and the historical case are shown together in the final panel.

To facilitate the understanding of this complex system, the results presented here (Figure 2, Figure 3, Figure 4) have been grouped (i) under an overall summary per strategy, (ii) by major theme (ecological, economic and social) and (iii) with the trade-offs between strategies (and overall comparative system-level results) provided at the end (Figure 5). These analyses generated an extensive set of results in terms of stock and broader ecosystem status, industry performance, social outcomes, management effect and cost. The volume of results is beyond what can be reported fully here so only a summary of the results will be given. A more detailed presentation of the results can be found in [24].

### Summary of the management strategy outcomes

Under the 2003 status quo management strategy, effort remains at around recent observed levels (Figure 2D), and vessels push into more marginal areas as the fishery tries to improve its economic status. Within 9–12 years (depending on the fleet sector) economic pressure causes vessels to exit each of the major sectors – consistent losses (costs exceeding the value of the landed catch) year on year means they cannot service their debts. This reduction in fleet size is associated with a temporary recovery in total landed catch (Figure 2E), CPUE (Figure 2F–G) and profits (Figure 3H), but this recovery dissipates quickly. There is also a shift in targeting as traditionally targeted fin-fish resources become less lucrative and the sectors begin to target both higher trophic level chondrichthyans and lower trophic level squid and small pelagics. This leads to a slowly degrading ecosystem state (Figure 4), which takes many decades to recover, and poor public perception of the fishery (Figure 3E).

The enhanced quota management strategy sees effort remains at about the level observed historically for about a decade (10–14 years depending on the sector) before economic pressure forces vessels out of the major sectors (Figure 2D). As for the 2003 status quo case, this is due to consistent losses. The spatial management makes it difficult for deepwater fleets to be profitable, so they change their targeting and shift to shallower grounds. Landings remain at around historic levels until the target stocks (e.g. tiger flathead and gummy shark) are depleted past their target reference points and TACs are reduced accordingly (Figure 2E). While the harvest strategies act to slow depletion, they do not prevent it completely as the rules have been defined based on single species assessments and are stretched by the multispecies interactions and changing effort dynamics occurring within Atlantis-SE. The use of TACs as the dominant management measure means that overcatch (when landed catch exceeds the TAC for a species) is a problem with this strategy. Towards the end of the projection period, when many traditionally targeted groups have constraining TACs and lower CPUE (Figure 2G), there is some target shifting to the previous un- (or only lightly) exploited chondrichthyans, as well as shallow demersal and forage fish and squid. Ultimately this activity leads to strong economic performance for the trawl sectors under this management strategy. The non-trawl sectors show variable performance, making strong gains when fleet sizes drop, but declining again once their main target stocks are depleted. This boom-bust cycle is reflected in port activity (Figure 3F), public perception (Figure 3E) and the ecological status of the shallow system components. In contrast the status of the system is fairly good for diversity (Figure 4A) and stocks in the deeper waters.

In the case of the integrated management strategy there is an immediate shift in the system, with all sectors contracting in size after 4–8 years. Concomitantly, the footprint of the entire fishery is confined to fishing grounds in the eastern Great Australian Bight, around Tasmania and off southeastern Victoria. Landed catches generally stabilise at a level lower than that taken historically (Figure 2E). While TACs can be strongly constraining, it is not unusual for the TAC of a target species to go unfilled due to the lack of quota for a byproduct group. Overcatch and high grading of constraining species remain an issue, while the broad use of spatial management highlights that without sufficient movement between reserves and open grounds it is possible for the available fish to be depleted even when the bulk of the population is doing well. Nevertheless, overall the majority of the performance measures (including average size of the catch, CPUE, profitability, public perception, and the biological status of the majority of groups) are stable or increase through time (Figures 2–4).

The conservation-dominated strategy depends on the extensive use of spatial management (that effectively closes >80% of all fishing grounds), which leads to solid conservation-based performance (Figure 4) and four-fold increases in CPUE for key target species (Figure 2G). However, this comes at a significant industry and human cost. High levels of competition on fishing grounds that remain open, with little scope for effort displacement, leads immediately to a drop in effort and catches (Figure 2D–E) - even before a scheduled buyback. Despite the exit of many vessels from all major sectors of the fishery within 5–7 years, the majority of the boats remain tied up in port for much of the year, living off any existing savings. Moreover, while fishing trips are substantially shorter (Figure 2H), this does not translate into drastically reduced costs (Figure 3D,G). Ultimately the fisheries are not profitable (Figure 3H), which leads to a significant downturn in the port economies – port population sizes dropping by 15–20% or more (Figure 3F). The buyback is ineffective as it simply removes those boats that were effectively already non-participants in the fishery. The drop in effort it produces is therefore negligible (Figure 2D).

### Industry performance

The summaries given above hide a multitude of detail. For instance, there are differential impacts of the management strategies across fleets. Under 2003 status quo and enhanced quota management, the 24–28% reduction in overall effort (Figure 2D) masks an increase in the activities of the Great Australian Bight Trawl (GABT) fishery, which grows steadily through time so that by 2020 its effort has increased by 27–42%. However, the conservation dominated strategy leads to a more ubiquitous reduction in effort (with all sectors dropping by 50% or more).

Changes in effort are associated with changes in fisher behaviour and trip length (Figure 2H). Under the 2003 status quo and enhanced quota management strategies, fishers make increasingly long trips, passing through multiple fishing grounds and fishing as they go. The exception to this is the deepwater trawlers who switch from depleted deep-water stocks to seasonal targeting of the most productive grounds on the continental shelf. This shift undermines the efficacy of the management system for species such as tiger flathead, where any initial benefits from the management restrictions is lost long term. There are also significant changes in fisher behaviour under the other two management strategies. The extensive zoning used in the conservation dominated strategy prevents a broad fishery footprint – seeing more effort on the outer shelf rather than on the deeper reaches of the upper slope (Figure S4), which are not economically viable given the spatial management arrangements. Ultimately the fishers in this scenario begin “pulse fishing” – where intense pressure is put into a few trips per year, during months when expected market prices are highest, with the operators attempting to minimise costs by remaining tied up for long periods in between (Figure S5). Fishing is more temporally consistent under integrated management, but the effort displacement driven by closures leads to a mosaic of intensively fished grounds and protected areas (particularly off Victoria and Tasmania) resulting in shorter trip lengths (Figure 2H), with boats tending to travel directly to and from preferred grounds. A significant number of operators also effectively become quota brokers (maintaining only low level of effort and leasing their quota), which allows the most efficient operators to increase their effort as resources free up and competition for access eases. Consequently, the effort reductions seen in this management strategy (of 16–37%; Figure 2D) are not as great as may be expected given that 18–65% of the boats exit the different fisheries sectors (Figure S6).

The shifts in effort are both driven by, and result in, changes in landed catch (Figure 2E). Under the high levels of effort in the 2003 status quo and enhanced quota management strategies, overall total landed catch continues the historic decline, despite temporary recoveries (by 25%) immediately following major effort contractions and the expansion of targeting to include less depleted fish groups. Both of these strategies display sequential depletion of stocks, shifting from demersal fin-fish to chondrichthyans and ultimately to invertebrates, especially squid (up by 166%), and small pelagics (increasing by 35%). Squid in particular is used as a means of subsidising the primary fishing activities of the trawl sectors. In contrast, the landing of small pelagics remains primarily the domain of the dedicated small pelagics sector. This sequential shift in targeting is particularly prevalent under enhanced quota management. TACs constrain the landed catch of many target groups under this strategy, but instead of the fishery reducing effort once quotas are filled, they move on to new targets that either aren't yet under the quota system or are effectively unconstrained by it; any take of groups that were once targets is then discarded. Consequently, this leads to high and variable discard rates under this strategy (Figure 2A). In contrast there is a 33% reduction in the volume of discards under the 2003 status quo management (Figure 2A), because as larger size classes become harder to find, more of the small sizes that were once discarded are retained.

Discards are low for the conservation dominated management strategy (Figure 2A), but there is still an increase in the landings of species taken by midwater trawls, jigs, and purse seines (as there are few spatial constraints on gear that does not interact with the bottom). In contrast, problems with discarding are not solved under integrated management. The use of spatial management and companion TACs (where the quotas of species often caught together are linked) constrain the catch of many species, with the lack of available quota for one species potentially leading to significant undercatch of quota for other companion species (e.g. the lack of available spotted warehou quota leads to 60–75% of the potential ling catch being left uncaught). The magnitude of ‘lost catch’ (and potentially high discard rates) may be an overstatement, as the model does not have the ability to adapt fine scale targeting to the extent real fishers can.

### Social and economic outcomes

Given 2003 status quo management was a continuation of the management regime in place in 2003, it is not surprising that under this form of management the access to the fishery and management stability are unchanged (Figure 3A–B). The fisheries' social licence is low, however, due to the poor ecological state and the influence of lobbying, negotiating and compromise on the setting of TACs (Figure 3E). This bleak outlook is reinforced across the economic indicators, with only marginal profits (Figure 3G–H) and low rates of investment. The economic situation is better under enhanced quota management (with profits of about $1000/t across the fishery), but the social licence is still low (Figure 3E), due to the pressure on the ecosystem, drop in port populations (Figure 3F) and low rates of investment. Moreover, this strategy sees high management costs due to the large number of groups under quota management (Figure 3D). As the SESSF is a cost recovery fishery, this has strong implications for the viability of the fishery. Costs of trading and leasing quota, especially for byproduct groups (non-target species of sufficient value to warrant landing them), also become a significant on-going concern for fishers.

The greatest increase in the gross value of product and profitability (Figure 3H) occurs under integrated management, especially for the trawl fleets, it is more marginal for other gears where costs are 2–3× higher. The increase in profitability is realised once fleet sizes have been reduced, with average profits rising to about $4000/t landed. The profitable nature of the fishery is reflected in relatively high levels of port activity (which drops by <7% from historical levels under this strategy, compared to >20% drop under the other strategies) and higher levels of investment long term. Nevertheless, access to the resources is highly constrained (Figure 3A) and there are also strong transient costs (of nearly $9 million per year in management costs) associated with the introduction of the various components of this integrated management strategy.

The synchrony of the social and economic state of the fishery and the health of the ecosystem seen in the first three strategies is not evident for the conservation dominated strategy. High management costs associated with maintaining the network of spatial closures ($7.7 million per year), in combination with the substantial reduction in fleet size, sees management costs per boat rise by more than five-fold (Figure 3D). In contrast, trading costs remain low, as the small amount of landed catch means quota is readily available (Figure 3C). Overall however, the low volume of catch means that GVP is never high (at about $30 million compared to about $95 million under 2003 status quo and enhanced management and $125 million under integrated management), which means that it is hard (and often impossible) for vessels to get sufficient catch to cover daily costs.

### Ecological outcomes

Without the management constraints present in the other strategies the impacts on the ecological system under 2003 status quo management are strong. For example, habitat interactions are initially higher than in any other strategy (Figure 2B). Furthermore, the unconstrained fisheries under this management strategy lead to further decline (by 35–65%) in nearly every target group (e.g. demersal fish complex in Figure S7), as well as in diversity (Figure 4A–E). Only scavengers benefit to any substantial degree from the fisheries activities under the 2003 status quo management strategy, as the amount of detritus (fed by discards, incidental mortality and feedback in the detritus-based food web) grows steadily through time (Figure S7), providing a significant food resource for these groups. In turn, this exacerbates the changes caused by direct fisheries interactions, leading to stronger restructuring of the system (e.g. the microbial and detritus webs grow threefold).

Under enhanced quota management habitat interactions and the skewing of the size spectra due to depletion of large fish are not as severe as under 2003 status quo management (Figure 4F–G), though there can be localised declines of as much as 61%. The management restrictions do see an improvement in the status of species of conservation concern (e.g. seabirds and marine mammals, the abundance of which nearly double or more; Figure 4D, Figure S7), but they fail to prevent a 30% decrease in deepwater sharks, which are due in part to long-term ecosystem processes that are still reacting to changes that occurred or began during the historical period.

The ecological state of the system is improved under integrated management. The combination of management measures minimise habitat interactions (Figure 2B) and sees diversity increase (Figure 4A). Under this strategy there is a recovery of the majority of the threatened and endangered species (Figure 4D) and all of the target species (Figure S7) – except gulper sharks and orange roughy – stabilise at about their target reference points. Under the conservation dominated management strategies these ecological outcomes are even stronger, facilitated by an almost complete lack of interactions with biogenic habitat (Figure 2B), and threatened and endangered groups (Figure 2C). Only forage groups (Figure S7) or scavengers show declines (by 10–28%), due to increased predation pressure and a drop away in the detritus based (discard fed) food web.

### Comparative results

The management of complex systems ultimately comes down to trade offs between objectives related to different components of the system (social, economic or ecological) and defined by different stakeholder groups. One way to highlight the form of these trade offs is to present them explicitly. To this end overall aggregate performance measure kite diagrams are shown here for the last historical year and the final year of each management strategy simulation (Figure 5). All strategies have a similar ecological state through the early years (reflecting past impacts) then diverge quickly leading to a diverse range of values for economic, social and management measures. The 2003 status quo strategy appears to have a relatively strong performance with regard to certainty about management access and stability, but this is an artefact of the failure of this simple measure to distinguish improved access and stability due to improved system state and management effectiveness from operators comfortable with a known ‘dysfunctional’ system. With respect to the other performance measures (social, economic, ecological and industrial) the poor performance of this strategy is clear. It does provide high short-term economic gains, but in the long term resources are exhausted, resulting in ecological and therefore economic deterioration in the system. In particular, the relative performance measure for industry is less than half of that under any other strategy.

The application of historical ‘best practice’ management approaches based around transferable quota schemes does see the enhanced quota management out-perform the 2003 status quo strategy in terms of the industry indicator, though economic performance is mixed as high effort levels result in high costs. Management overheads are also high in this strategy due to the relative instability of TACs, though in terms of the other measures the enhanced quota management provides a slight improvement on the 2003 status quo strategy ecologically and socially.

The integrated management strategy, in contrast, shows a relatively even performance across all performance indicators, and, although it does shift pressure to more productive shelf stocks, in general it performs well. This is not the case for the conservation dominated strategy. In this case the management overheads, certainty about access and ecological state are high, but this is overwhelmed by very poor economic outcomes (leading to the lopsided shape of the kite diagram in Figure 5).

## Discussion

### Efficacy of management measures - the need for integrated management

While the work presented in this paper focuses on a case study in southeastern Australia, the multi-sector, multi-species nature of the fishery means that it has general applicability. Australia, like many nations is struggling with what a practical form of EBFM will look like. EBFM often means different things to different people, nations and institutions. Over the last decade the scientific, political and societal push for the uptake of EBFM has grown [3], [5], [27]–[28], with some acknowledgement that human dimensions must be considered as an integrated part of such an approach [6]–[7], [29].

A focus on integrated management is prominent in the Australian Ministerial Direction of 2005 (available at http://www.daff.gov.au/fisheries/domestic/fishingfuture) that reshaped the management of the SESSF following the rethink that was informed by the work presented in this paper. Discussions regarding the final form of the restructuring were supported by information from the earlier stages of the project; ultimately, the restructuring saw the fishery take on a form of management based on a modified version of the integrated management strategy explored here. Interestingly, the integrated management strategy was so far removed from the management practices prior to 2005, and the alternatives under serious discussion by stakeholders at the time, that it was originally referred to as the ‘blue sky’ strategy. However, by the time the project finished the management had shifted to the point that many features of integrated management had become almost business as usual. This outcome clearly demonstrates that workable solutions to EBFM do exist and that to address the multitude of objectives involved means that EBFM will need to be an integrated mix of many forms of management (a balanced combination of a variety of input, output and technical management measures); individual classes of management actions will not suffice.

Each of the management strategies explored here proved to have its own set of strengths and weaknesses (Table 4), with no single strategy dominating on all aspects of performance. Overall however, the integrated management strategy had the fewest shortcomings; it did not produce the “best” result against all objectives, but it consistently preformed well and it avoided the pitfalls that individually (and potentially catastrophically) marred the performance of the other management strategies. This is in direct contrast to much of the literature over the last two decades which has focused on individual measures, such as individual transferable quota or spatial management, as the “best way” of achieving successful long-term fisheries management (e.g. [12], [17]). The results of this modelling study indicate that neither of these strategies provides a comprehensive solution in itself.

**Table 4:** Summary of strengths and weaknesses of each management strategy.

Strategy	Strength	Weakness
Status Quo	Short term economic returns	Extended effort footprint and high absolute level of effort
	High absolute catch	Long-term economic decline
	Low management costs	Fleet collapse
	Fishers know how the management system works (i.e. it doesn't so there will be no new changes)	Long-term deterioration of biological system (and poor diversity)
		Low GVP
		Poor CPUE
		Discards remain unconstrained (and potentially high)
		High TEP and habitat interactions
		Poor social perception
		Little if any investment
Enhanced quota management	Short term economic returns	Extended effort footprint and high absolute level of effort
	GVP in short to medium term	High costs (including management costs into the long-term)
	Deepwater biomass recovers	CPUE low in some sectors
	Diversity recovers in some areas	Long-term GVP
	High absolute catch	Discards remain high
	Moderate habitat interactions	High number of TEP interactions
	Some reduction in gear conflict	Shelf and upper slope biomass heavily impacted
		Poor social perception
		Sensitivity to the form of non-quota management measures (without them there is poor long-term ecological and economic performance)
Integrated management	Reduced effort footprint and moderate levels of absolute effort	High short-term disruption associated with transition in fleet size and structure and new management arrangements
	Economic health of all sectors improved	Pressure on productive shelf stocks
	Widespread improvement in biological system state	Discards remain a potential problem
	Reduced habitat interactions	
	Reduced gear conflict	
	Moderate levels of absolute catch	
	Higher CPUE	
	GVP and profits	
	True management stability (i.e. management occurring and stable)	
	Moderate management costs	
	Smooth transition in fleet size and structure (no collapse)	
	Improved social perception	
	Investment in the industry and steadily increasing returns on that investment	
Conservation dominated	True management stability (i.e. management occurring and stable)	Poor economic returns (fishery not economically viable in long-term)
	Reduced footprint and absolute level of effort	Poor return on investment
	High CPUE	Poor GVP
	Discards reduced substantially	Low catches
	Widespread improvement in biological system state	High per boat management costs
	Substantially reduced TEP and habitat interactions	High short term research costs
	Reduction in gear conflict	High short-term disruption associated with new management arrangements
	Improved social perception	

The results of the strategies that focused on quota management (including use of ITQs) indicate that incentive structures can undermine the intent of a quota-based (TAC) management system. The various dynamics that undermined the efficacy of quota management in the simulations (e.g. lags in quota setting, non-constraining quotas, unaccounted for sources of mortality, high grading and over-catch, and ecological interactions) are in line with those found in empirically based reviews [18]–[19], [30]. Without universal observer coverage, instead of withdrawing once quotas are filled, vessels may shift their targeting to species that are either not under the quota system or are unconstrained by it (e.g. squid), and discard any catch of species that have exceeded quota levels. Encouragingly, work from Canada shows that the response of fishers, in the British Columbia groundfish fishery, to 100% observer coverage saw the realignment of incentives and rapid modification of behaviour. This in turn led to substantial improvement in targeting of specific species, reduction (and avoidance) of bycatch [18], [31] and increased cooperation amongst the vessels.

Other economic or incentive based management options, such as gear switching and buybacks, can be expensive and may not be as effective as expected due to the foibles of human behaviour [32]–[33]. In the simulations, gear switching was not a viable solution to autonomous restructuring, as there was little uptake unless it was heavily subsidised. The infrastructure and quota costs of using alternative gears proved prohibitive without subsidies and even then it acts as a stopgap – operators postpone the inevitable by initially switching gears, but ultimately the vessels ended up leaving the fishery anyway. Poorly timed buybacks can also lead to a dissipation of any potential benefits – this occurs when they are implemented too early (before there is significant economic decline) or too late (when the system is in a very poor state both ecologically and economically).

The findings from this model study suggest that targeted spatial management is an indispensable part of any successful management strategy for a multifaceted and complicated fishery such as the SESSF. Some objectives (e.g. conservation of sensitive habitats) can only be achieved via zoning. Nevertheless, spatial zoning is not free of its own unforeseen consequences. As has previously been found by other researchers (e.g. [24], [34]–[37], the simulations indicated that small spatial coverage has little overall benefit for fisheries; closures do not reduce effort by themselves; and the impact of spatial management will vary across species and fleets – often in relation to their mobility and trophic position (prey species do not see the same benefit that predatory species do). The potential economic collapse of fisheries in systems with extensive closures has received less attention, as many economic analyses of MPAs have focused on the benefits for tourism [38]–[40].

### Trade-offs

Complex trade-offs exist when trying to satisfy the various ecological, economic and social objectives at the heart of EBFM. The simulated management strategies explored here highlighted a number of important trade-offs: between conservation objectives and economic returns; short term costs and long term payoffs (or short term profits and long term risk); economic cost structures (smaller for smaller vessels) and social mobility and adaptive capacity (which are smaller for owner-operators tied to home ports by family and community ties); reduction in competition between operators (as fleet sizes contract) and the per vessel management cost burden in a cost recovery fishery. The simulations also show how trade-offs between system components can lead to unanticipated outcomes of management decisions. For instance: targeting shifts from deeper water groups to major shelf species, or from primary to secondary target species, as fisheries are impacted by management constraints and rising costs; and the use of companion TACs lead to the ecological decline of weaker stocks or significant economic costs (if the catch of productive stocks is highly constrained).

Trade-offs are synonymous with EBFM, not only between objectives, but also between system components. Actions aimed at supporting outcomes for one component could undermine the status of another component (as was seen in the strategies used here, when management introduced to protect deep water stocks undermined the status of shelf species) unless a system level understanding is embraced from the outset. Achieving such an alignment of management intent and outcome will require a balanced and integrated set of management measures, as management objectives and industry incentives will be more likely to be aligned across social, cultural and economic drivers.

### Appropriate use of end-to-end models

Models with the degree of process detail included in Atlantis-SE are associated with high degrees of uncertainty. This makes them unsuitable as a tool for informing tactical management decisions, such as setting catch quotas or determining best location of closed areas, which are best left to specialised models such as fishery stock assessment models (e.g. [41]) and MARXAN [42]. However, when used appropriately – for example, in ensembles and in frameworks like management strategy evaluation which directly embrace uncertainty (discussed further in the Text S1) – system-level models such as Atlantis can provide a sound basis for informing whole-of-system management strategies that provide integrated rather than piecemeal solutions to complex fishery management problems. However they also represent a large investment in scientific resources, and therefore it is unlikely that models of this type will be developed for a large number of fisheries. It is therefore important that lessons learnt from case studies, such as the SESSF example presented here, are carried over to other fisheries and systems [21]. It is equally important to realise that the kind of management strategy evaluations discussed in this paper should not be seen as a strict forecast or assessment of the fishery or exploited stocks. At the system-level these methods should only be used to give strategic insights into the consequences and potential trade-offs associated with management of natural resources. The results of these evaluations do not provide optimised or prescriptive management advice; they do however provide strategic decision support.

The best use of these models hinges on defining and testing a tractable set of plausible and feasible alternative management strategies. The large number of variables and processes in a model such as Atlantis means that there is an enormous number of possible management options that could be tested, many more than could ever be effectively analysed or usefully considered. The key to successful application of MSE using a model such as Atlantis is to consult repeatedly (iteratively) with a broad range of stakeholders (e.g. fishers, company owners, managers, scientists, eNGOs, economists, retailers and other users of the resource such as recreational fishers or community members), who drive the process and help define a tractable set of plausible and feasible alternative management strategies. This may require some strategic input from the MSE analyst as those closest to the fishery may initially need encouragement to broaden their vision, as happened in this case around the ‘blue sky’ (integrated management) strategy. This iterative approach to identifying a tractable but diverse set of options may also be achieved using simpler, more qualitative approaches to MSE. In the study described here, initial options were identified and explored using such an approach [43]. This proved to be an effective engagement mechanism for stakeholders and resulted in the range of strategies then tested more formally using the Atlantis model described in this study.

## Conclusions

Fisheries (and marine natural resource management more generally) are moving towards an ecosystem approach and system level management. Both empirical experience (e.g. the fate of fisheries such as that for southern bluefin tuna [44], Norwegian cod fishery [45] or the Northern Cod stock in Canada [46]) and modelling studies such as the one presented here reinforce that focusing on individual management measures and simplistic strategies will be inadequate to meet the ecological, economic and social objectives that must be addressed for successful ecosystem based management. Our results show that successful system level management is possible and that it can be jointly uncovered by bringing together options proposed by all parties with a stake in fisheries – industry, conservation, community, managers and scientists. The particular measures adopted in effective integrated packages of management measures will almost certainly vary from one ecosystem and fishery to the next and will need to evolve through time along with the systems they seek to manage. However, when such system-level options are adopted, they can lead to rapid changes in fisheries resulting in more effective achievement of social, economic and ecological objectives.

## Supporting Information

Figure S1 **Spatial management maps for bottom contact fisheries in the different management strategies.** (a) status quo, (b) enhanced quota, (c) integrated and (d) conservation dominated. Key indicates percentage of the box open to fishing. Bold line indicates the boundary of main longline fisheries. Minor fisheries could have further restrictions, whereas surface and midwater fisheries typically had fewer restrictions and could access much of the area.
(TIF)

Figure S2 **Comparison of catch per unit effort time series for Atlantis-SE versus actual historic time series for blue warehou.**
(TIF)

Figure S3 **Comparison of longline effort time series for Atlantis-SE versus actual historic time series.**
(TIF)

Figure S4 **Annual effort per depth stratum per gear type per management strategy.**
(TIF)

Figure S5 **Proportional distribution of trips per month across sectors in the final 5 years of each management strategy.**
(TIF)

Figure S6 **Proportion of boats per sector exiting the fishery under the integrated management strategy.**
(TIF)

Figure S7 **Relative final biomass for the major types of biological components under each management strategy.** Dark band shows interquartile range and lighter bands across all parameterisations and the lighter bands indicate the range containing >95% of the results.
(TIF)

Text S1 **Supporting Information.**
(DOCX)
