# Supplementary figures and images for: An Integrated Approach Is Needed for Ecosystem Based Fisheries Management: Insights from Ecosystem-Level Management Strategy Evaluation
Source: PLoS One. 2014 Jan 13;9(1):e84242. doi: 10.1371/journal.pone.0084242 (PMC3890272; doi:10.1371/journal.pone.0084242)

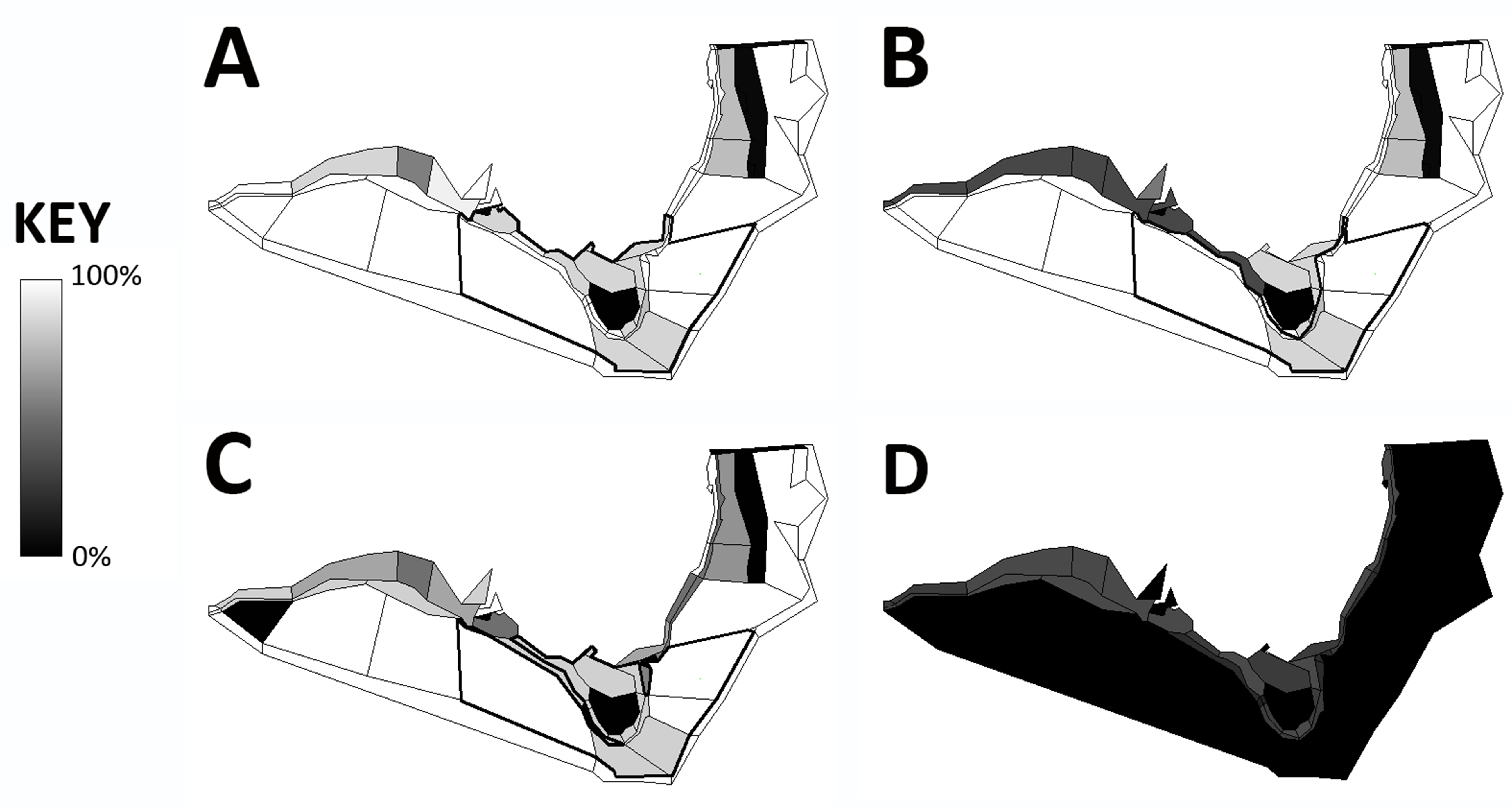

Supplement: Figure S1 — Spatial management maps for bottom contact fisheries in the different management strategies. (a) status quo, (b) enhanced quota, (c) integrated and (d) conservation dominated. Key indicates percentage of the box open to fishing. Bold line indicates the boundary of main longline fisheries. Minor fisheries could have further restrictions, whereas surface and midwater fisheries typically had fewer restrictions and could access much of the area. (TIF) [file pone.0084242.s001.tif]

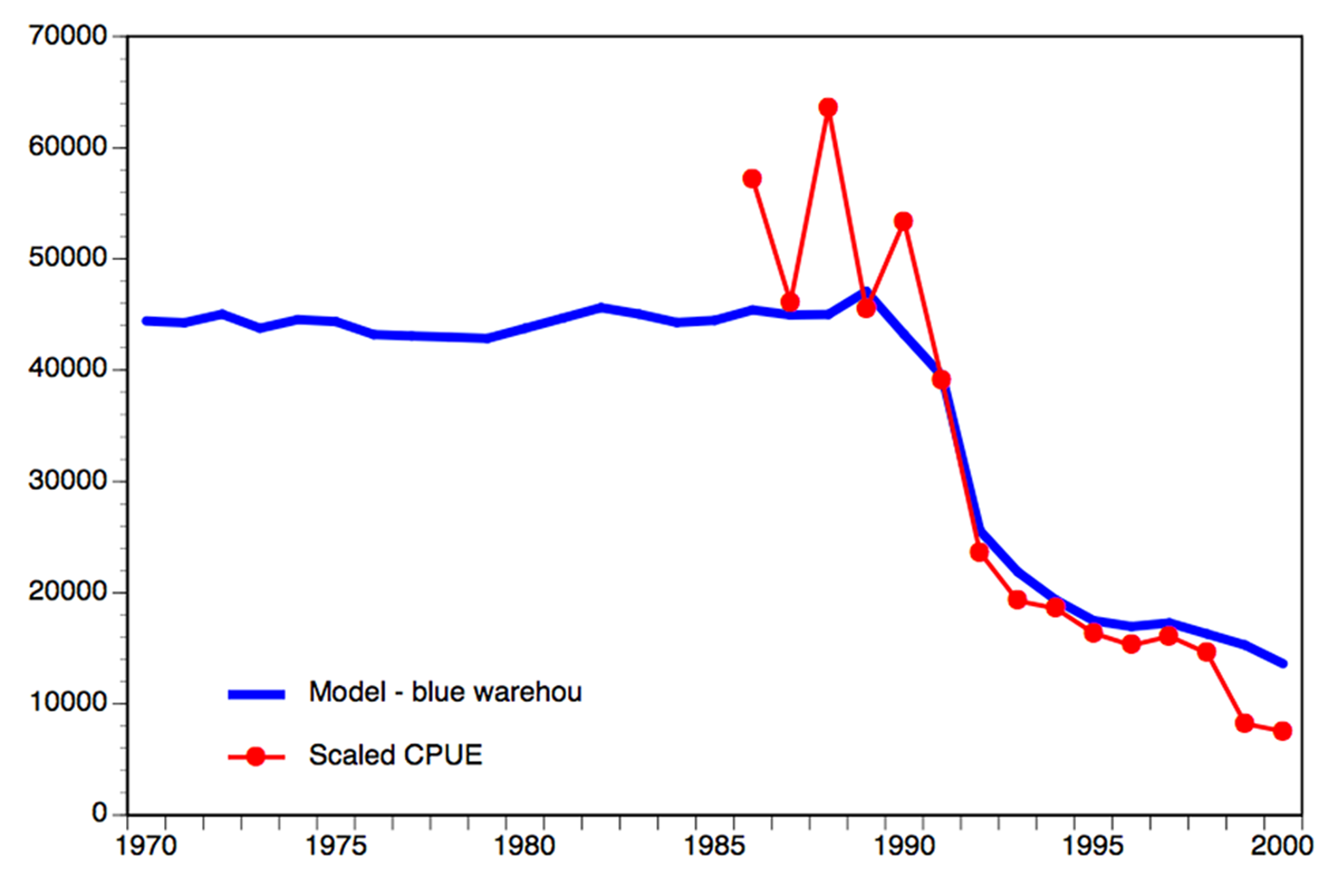

Supplement: Figure S2 — Comparison of catch per unit effort time series for Atlantis-SE versus actual historic time series for blue warehou. (TIF) [file pone.0084242.s002.tif]

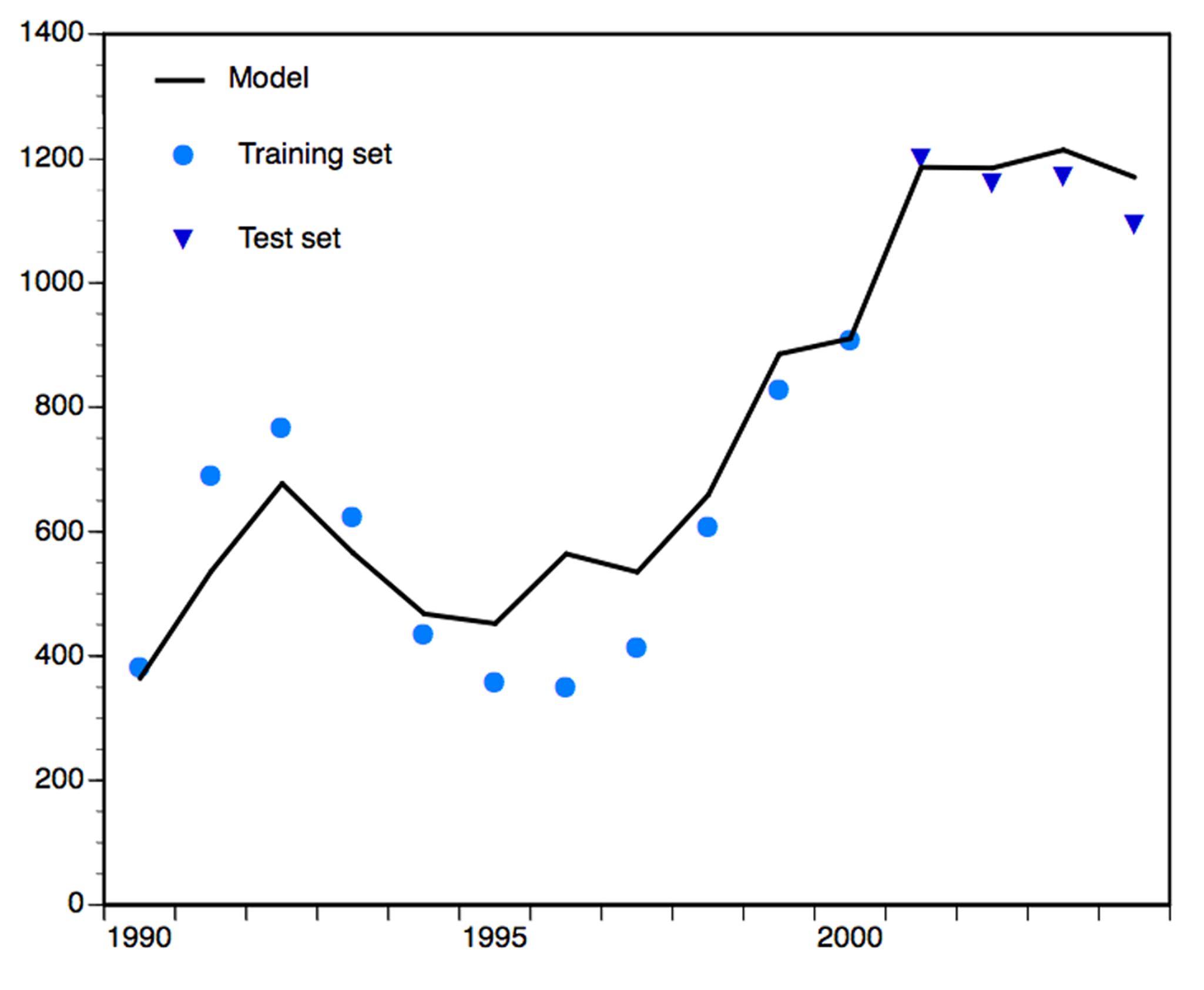

Supplement: Figure S3 — Comparison of longline effort time series for Atlantis-SE versus actual historic time series. (TIF) [file pone.0084242.s003.tif]

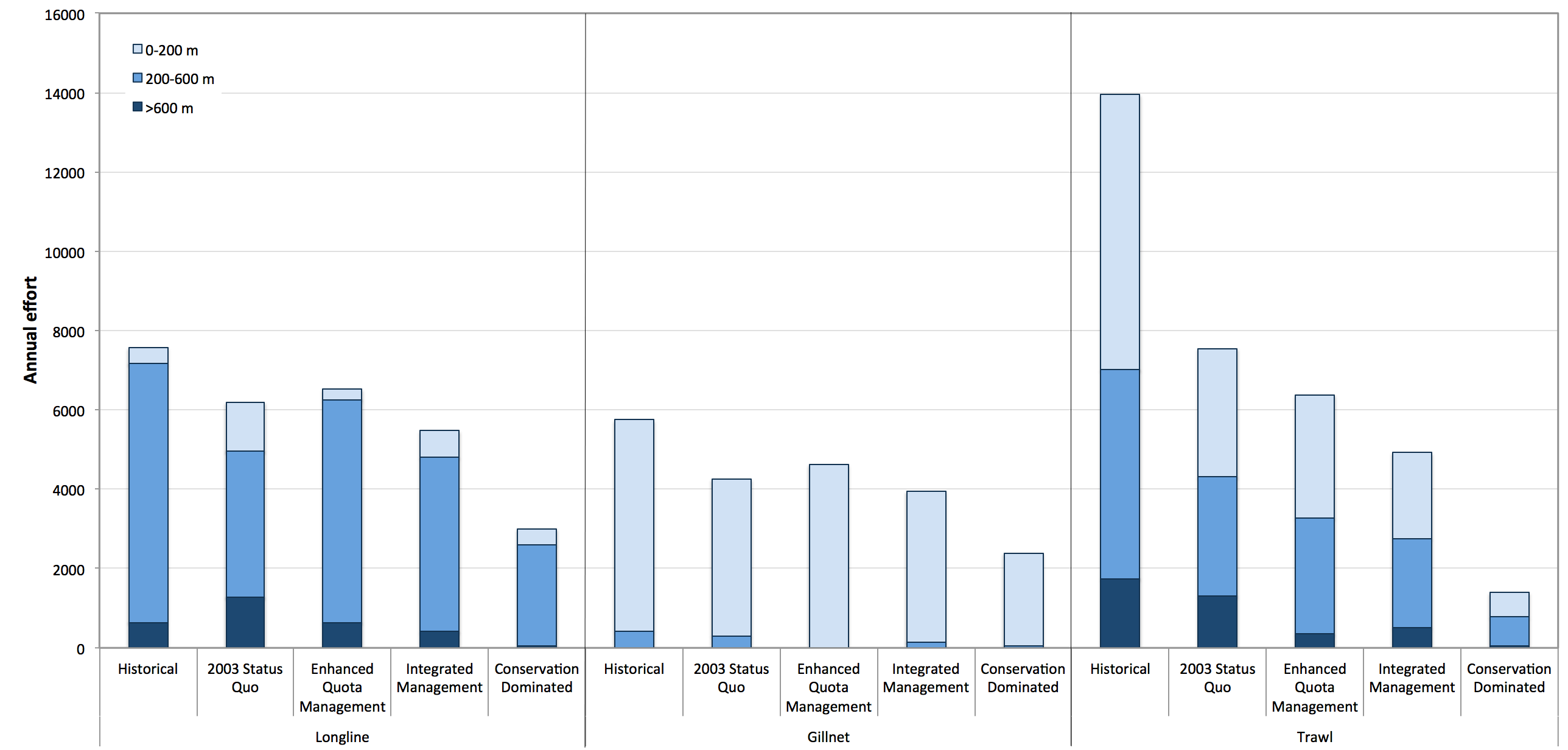

Supplement: Figure S4 — Annual effort per depth stratum per gear type per management strategy. (TIF) [file pone.0084242.s004.tif]

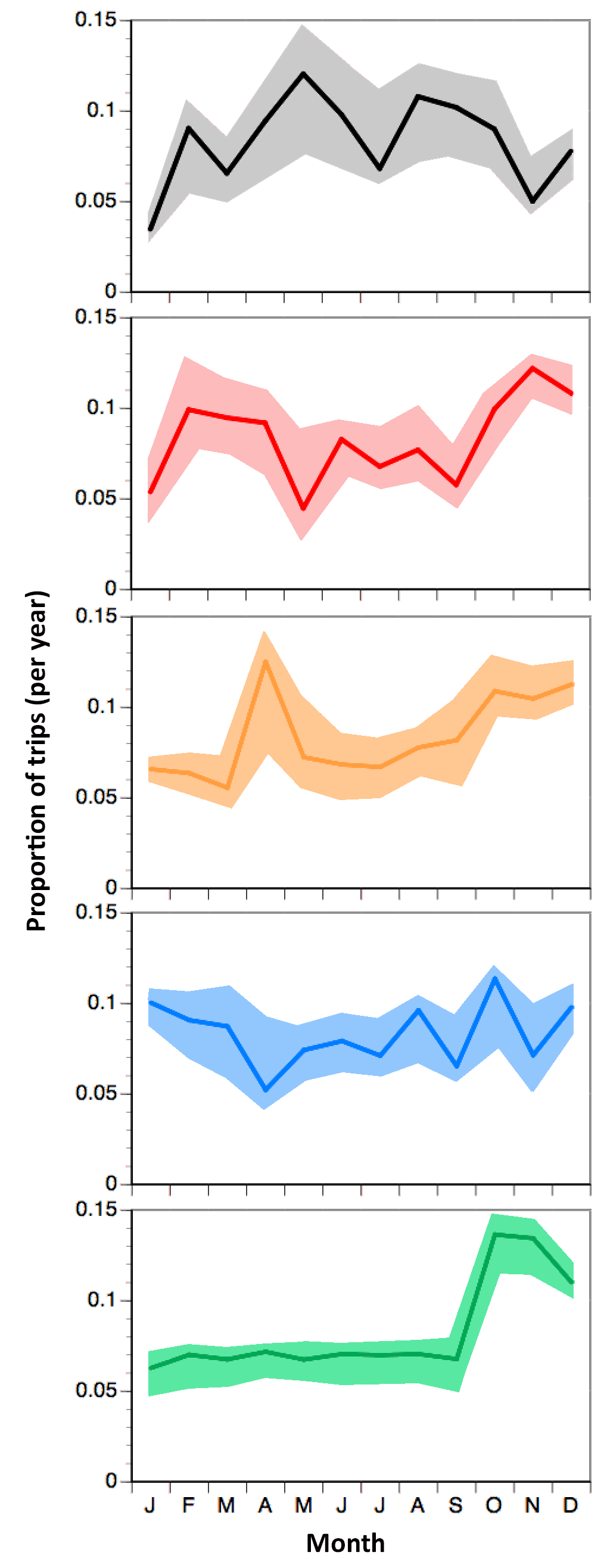

Supplement: Figure S5 — Proportional distribution of trips per month across sectors in the final 5 years of each management strategy. (TIF) [file pone.0084242.s005.tif]

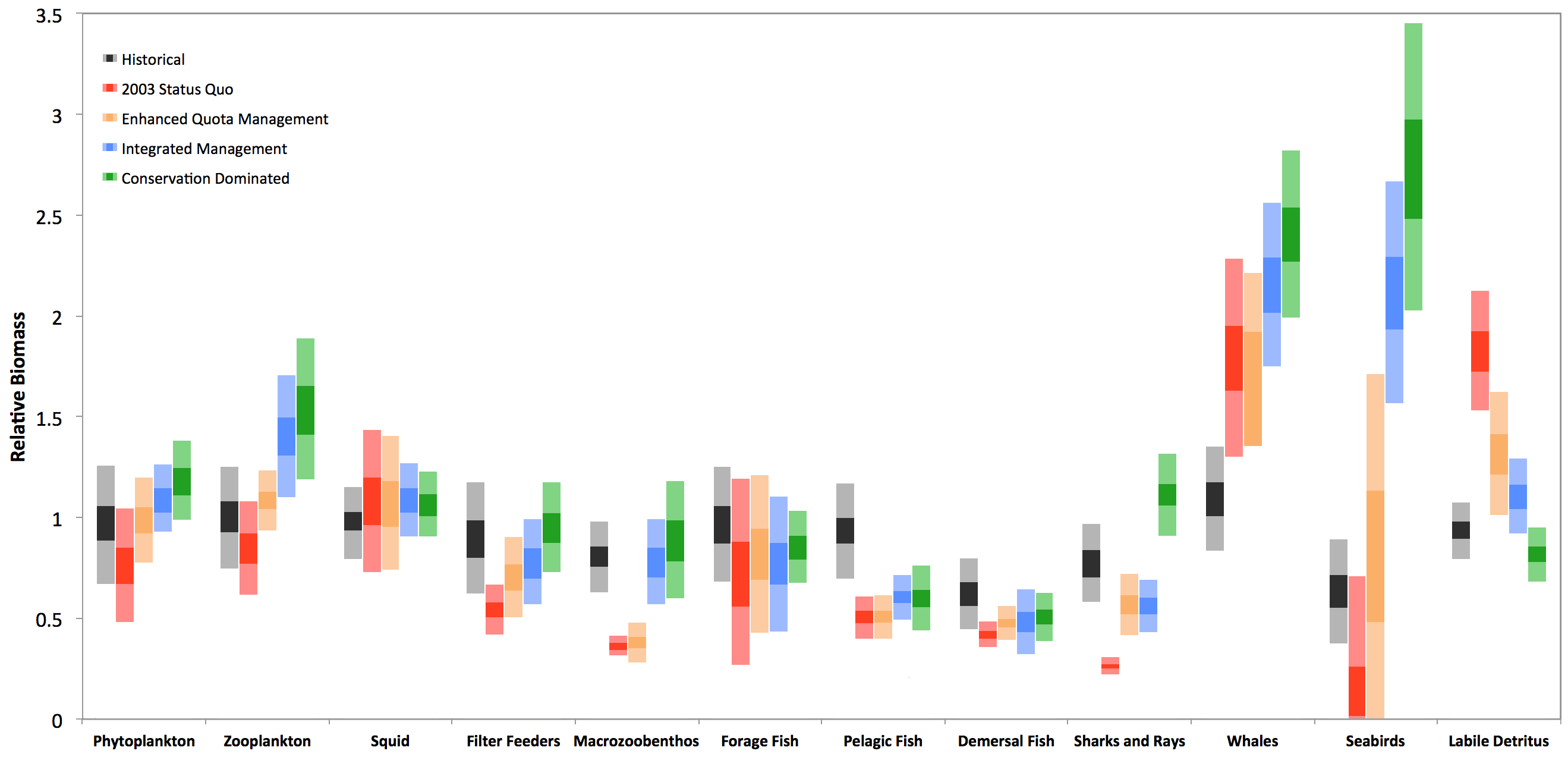

Supplement: Figure S7 — Relative final biomass for the major types of biological components under each management strategy. Dark band shows interquartile range and lighter bands across all parameterisations and the lighter bands indicate the range containing >95% of the results. (TIF) [file pone.0084242.s007.tif]
