# Supplementary material for: An Integrated Approach Is Needed for Ecosystem Based Fisheries Management: Insights from Ecosystem-Level Management Strategy Evaluation
Source: PLoS One. 2014 Jan 13;9(1):e84242. doi: 10.1371/journal.pone.0084242 (PMC3890272; doi:10.1371/journal.pone.0084242)
Supplement: Text S1 — Supporting Information. (DOCX) [file pone.0084242.s008.docx]

**Supporting Information**

**Atlantis model summary**

***Biophysical system***

The spatial domain of the model represents the southeast regional marine ecosystem, which covers 3.7 million km^2^ of Australia’s south eastern EEZ, from (117°48’ E, 46**°**51’ S) to (160**°**30’ E, 24**°**21’ S) (Figure S1). This region spans tropical, subtropical, cool temperate and subantarctic environments and includes: a wide variety of bottom types (e.g. from silts and oozes through to rocky reefs and exposed limestone bedrock); large bays and gulfs; coastal, shelf, slope and open ocean waters; and submarine features like seamounts and submerged canyons. Water properties in the area (for instance sea surface temperature, the strength and location of upwellings, the supply of nutrients) and the productivity and distribution of the biological components of the ecosystem are all heavily influenced by the Zeehan and East Australian currents. Ecologically, the area includes some of the most productive of Australia’s waters – the seasonal upwellings off the Bonnie coast (in South Australia) and Tasmania see them classed as >300 gCm^-2^yr^-1^ LME components [S1]. These waters are also highly diverse, containing a large number of highly migratory, relic and endemic species [S2].

Capturing this complexity is quite challenging and we chose to develop an implementation of the Atlantis modelling framework. The resulting model, Atlantis-SE, is a three dimensional box-model that explicitly represents the major biophysical processes in the system (currents, nutrient cycles, primary production, consumption, growth, waste production, movement, reproduction, major stock structure and the influence of environmental drivers). The physical environment features (current flows in the form of bulk exchanges, temperature and salinity) were estimated using the Commonwealth Scientific and Industrial Research Organisation (CSIRO) 0.1° x 0.1° ocean forecasting model (OFAM) [S3]. A table of ecological groups included in the model is given in Table S1 in Text S1. Full specification of the parameterisation of the model is beyond the scope of this paper and further details of the way in which the biophysical complexity of the region was represented in the Atlantis-SE biophysical system can be found in [S4].

***Fishing fleets***

There is a wide range of fisheries in the region, of different sizes, under different jurisdictions (State and Commonwealth), using different gears and targeting different parts of the ecosystem. The representation of this diversity of methods was based on targeting and gear use. All fisheries (including state fisheries) were grouped into fleets, further divided into “fleet components” and sub-divided further based on vessel size (if there was a wide range in fleet composition at that level). The Commonwealth fisheries were the focus of the study and so were represented as dynamic fleets (using the social and economically driven fleet dynamics model detailed in [S4-S5]. In contrast State fleets were aggregated more heavily and their distribution, gear use and effort levels were set at a fixed level and in a fixed spatial distribution in all runs. Recreational fishing was represented simply as a flat rate per recreational fisher (applied only in coastal boxes). A list of all fleets and fleet components used in the model and their gear and primary target details are given in Table S2 in Text S1.

***Social and economic system***

There are four main parts to the current socio-economic model in Atlantis:

1. *Calculation of fisheries associated economic indicators*. Economic indices (e.g. gross value of the landed catch; costs and profit; return on investment and capital utilisation) were calculated based on relationships described in [S6]. The monthly average market fish prices (per group) were calculated using autoregressive models (where the next projected price is based on the previous price and a seasonal demand signal) based on species level data from the Melbourne (1992-2001) and Sydney (1992-2004) markets (see [S4] for details of the final regressions and the quality of their fit).
2. *Fleet and port tracking*. This keeps track of fleet sizes, investment and disinvestment, which dictates the port activity status (based on landings channelled through a port), which in turn dictates the local population size, recreational fishing pressure and coastal habitat degradation and pollution. The number of vessels was updated monthly – recording any vessels that were decommissioned, forced from the fishery by debt, sold in a buyback scheme, or switched to alternative fisheries; if spare licences existed, new vessels may also enter a fishery. Investment, disinvestment and switching between fleets are based on the model by [S7], which constructs a probability of leaving a fishery (or buying into a fishery if spare licences are available) based on long-term net returns versus short-term payouts.
3. *Effort allocation*. The dynamic spatial and temporal of effort by fleet was based on social and economic factors (described in detail in [S4-S5], but accounts for vessel capacity, updating expectations of catch of target and bycatch spatially and seasonally, quota holdings, market prices, regulatory and other costs (including onshore costs), and the level habitual behaviour and flexibility of the operators.
4. *Quota allocation and trading*. From year to year a simple proportional allocation of owned quota was used (any leases, permanent or temporary were dealt with after quota was allocated to the owners). The quota price per species per vessel was calculated using the quota price model developed in New Zealand by [S8]. Through each year, the need to trade quota is then based on relative cumulative catch (as well as expected catch) versus quota in hand. Packages of quota (mixes of different species) are proposed by buyers (leasors) and sought by sellers (lenders), with pairings based on the best matches (which can be influenced by friendship networks). Further details of the quota allocation and trading model can be found in [S4].

***Monitoring and assessment***

In 2004 fisheries independent monitoring was limited in the SESSF, consequently the monitoring scheme incorporated in Atlantis-SE consists of the fishery statistics that were collected and used in assessments in the SESSF at the time, including catch, effort and some discarding data (from observers). The actual catch and effort were stored in the fisheries model, but error was added to capture small amounts of misreporting and unintentional errors.

A “pseudo-assessment” was used to derive the estimates of biomass, fishing and natural mortality. This was done by taking the actual (Atlantis-SE biophysical model) biomasses and numbers lost to predation and fishing, and adding (normally distributed) temporally autocorrelated error to represent uncertainties associated with the assessment process. These “values with error” were subsequently used to estimate the reference points and recommended biological catches (RBCs) for use with the management harvest control rule to determine the quotas. In comparison with the use of a CAB assessment model [S9], this approach led to differences in the RBCs of less than 10%, but had a significant computation saving. As the assessment model was not the focus of this study this was an acceptable compromise.

The Total Allowable Catch (TAC) is set based on the RBC, taking into consideration other biophysical and social pressures. The way in which the RBC and TAC were set depended on the decision-making method employed in each management strategy (detailed below). The first method attempted to replicate the “negotiated TAC setting” that occurred during the period of the late 1990s and early 2000s and consisted of the following rules:

1. if the catch rates had been increasing for more than five years then the TAC was raised by 20%;
2. if the catch rates had been decreasing for five years and the current rates were below a critical level (set at 5% of peak levels, based on data from the period) then the TAC was decreased by 50%.

The second method of RBC setting uses a harvest strategy framework (HSF) matching that introduced to the SESSF in 2006 [S10], which assigns each quota species to one of four tiers depending on the amount of information available for that species and determines the RBC based on the rules for that tier (see [S10] for further details). The final TAC is then set based on the calculated RBC after (potentially) taking a number of other considerations into account:

- Level of any companion TACs (if these were in use the quotas were rescaled based on the ratios of the species in the catch and whether the objective was to avoid constraints on catch of the abundant species or whether the weaker species was being protected)
- Discards (the F rate is calculated including discards and expected discards are subtracted from the RBC to give the new TAC)
- Whether spatial management is being traded off against any TAC reduction
- Constraints on step changes in TACs, as mentioned above

***Management system***

Fisheries management in Atlantis is expressed by the adjustment of management measures, based on the results from the assessment (or pseudo-assessment) models. The principal assessments are those for target and quota species, but impacts on other components of the ecosystem (bycatch, threatened, endangered and protected species and benthic habitats) can also be considered explicitly. This is done via the use of triggers associated with key species and habitat indicators, which identify the need for management action when the estimated biomass or coverage of that group drops below a limit reference point of 20% of the estimate of unperturbed levels.

For the management strategies considered here, the management measures considered were:

- gear size (which impacts selectivity, accessibility and escapement)
- discarding
- spatial management (zoning, spawning, threatened species-based and total closures)
- seasonal closures
- trip limits
- quotas (including basket, companion and regional TACs; where regional quotas are based on the stock status in each region, with regions defined as for the SESSF, which originally defined them based on presumed genetic stock distributions; basket quotas are were a group of species (e.g. all demersal sharks) are grouped under a single quota rather than having individual quotas; companion quotas are where species typically caught together are paired and their quotas are then scaled based on that ratio, if a weak-link is used then the quota of the more productive stock is scaled down so the weaker stock is not over-exploited, whereas if a strong-link is selected then the quota of the weaker stock is scaled up so the catch of the stronger stock is not as constrained by potential bycatch)

Of these, quotas and spatial management were the most frequently used measures.

The costs of general management, research, compliance, monitoring and other (buyback) costs were tracked on an annual basis using the following equations:

$$K_{gen,t}=K_{gen,0}$$

$$K_{res,t}=K_{res,0}\cdot\left(\varphi{}_{Q,t}\cdot\kappa_{Q}+\varphi_{res,t}\right)$$

$$K_{com,t}=K_{com,0}\cdot\left(1+N_{Z}\cdot\kappa_{com}+\kappa_{TAC}\cdot N_{Q,i}\right)$$

$$K_{mon,t}=K_{mon,0}\cdot\frac{N_{Q,i}}{N_{Q,hist}}\cdot\frac{\varphi_{Q,t}}{\varphi_{Q,0}}$$

where *K_gen,t_* is general costs in year *t*; *K_res,t_* is research costs in year *t*; *K_com,t_* is compliance costs in year *t*; *K_mon,t_* is monitoring costs in year *t*; *φ_Q,t_* is the proportion of species under quota that are assessed (and have TAC updated) in year *t*; *φ_res,t_* is the contribution of fisheries ecological research to the overall research costs (including tagging and other spatial studies as well as life history research); *κ_Q_* the scalar of costs for each additional species assessed (set to 1.15 on advice from AFMA and CSIRO financial officers); *κ_com_* the cost scalar associated with each extra spatial management area to be enforced (set to 0.007 on advice from AFMA); *κ_TAC_* the cost scalar associated with each extra species included in the quota management system (set to 0.005); *N_Z_* is the number of spatial management areas implemented under the strategy; *N_Q,i_* is the number of species included in the quota management system for the strategy; *N_Q,hist_* is the number of species under quota management in reality in the year 2000. The initial values for the costs were provided for the major sectors by AFMA, based on the values from 2006.

**A note on uncertainty**

Model uncertainty is always a significant issue with ecosystem models. Unfortunately classical sensitivity analyses are inadequate (and potentially even inappropriate) for considering this uncertainty [S11]. At present the only pragmatic way of addressing uncertainty in such large models is to create an ensemble of models from the multiple parameterisations that comply with the historical fitting criteria [S4] and span the plausible range of biomasses and dynamics. These parameterisations were found using a simple implementation of pattern-oriented modelling (see [S12] for a more detailed description of the method). Previous factor analyses of Atlantis were used to identify critical parameters, which were then simultaneous varied and the resulting output judged against logbook data and scientific survey time series to find parameterisations that capture different facets of the system at different spatial and temporal scales. Ultimately, this process lead to 3 parameterisations for each system configuration (see structural uncertainty below); with one parameterisation the “best fit”, another approximating the lower bounds of observed system productivity and a final one fitted to the upper bounds of recovery rates and system productivity.

An aspect of model uncertainty that often receives less attention is structural uncertainty. Extensive work using qualitative and network tools (e.g. loop analysis) went into the developmental stages of the project to minimise potential impacts of this form of uncertainty (using the methods of [S13]). The regular coloration clustering method [S14] was used on all available trophic data to find plausible system structures, these were augmented by adding in trophic and habitat dependencies seen in neighbouring ecosystems or similar systems elsewhere around the globe. Loop analysis was then used to check first whether the level of trophic aggregation effected the expression of potential perturbation effects (i.e. did it nullify or reverse the direction of change observed in less aggregated network structures). Finally the three most plausible system structures that showed the greatest range in perturbation responses (without introducing aggregation artefacts) were run forward through the entire MSE.

Within the MSE approach this handling of uncertainty is sufficient given that relative performance of management strategies is under consideration, not absolute values of specific outputs. The confidence in the veracity of the results in this case comes from whether or not a similar ranking of results is seen across the various model implementations and uncertainties. In this case, irrespective of the model variant used or the absolute magnitude of the results, the rank order of management strategy performance did not differ for the various indicators. The separation between the performance of the different strategies did expand (contract) between variants, but the rank order and final conclusions remained largely unchanged. The particular variants where there were some minor changes in the ranking are beyond the set of strategies considered here and are described in detail in [S4]. Given the consistency in the results, for clarity of plots show the range of outcomes across the ensemble of runs (multiple parameterisations for each system structure) with the discussion of the results focusing on the “best fit” parameterisation of the most likely system structure.

**Performance versus Historical Data**

Long-term fisheries independent time series do not exist for the southeast region of Australia. Consequently the model’s performance in comparison to observed time series had to be judged against historical fishing time series – effort and catch-per-unit-effort (CPUE). These data sets were available for the various fleets used in Atlantis and for the main target species. In all cases the fit to standardised CPUE series was quite good (e.g. Figure S2), except for Blue Grenadier, where the very large annual variability seen in reality (due to exceptionally strong occasional recruitments) could not be matched. The model also reproduces the gross patterns of effort per year reasonably well for both training and test data points (Figure S3). However, it tends to run along trend lines and underestimate the true size of peaks or troughs that deviate from that trend. The model line also tends to overstate effort levels (by 15-25%) during the earlier years of the time series for the demersal trawl fisheries, but does not show such bias for other gear types.

**References**

S1. Anon (2008) LME Information Portal - LMEs of the World NOAA LME Program Office, <http://www.lme.noaa.gov/>

S2. Coleman N, Cuff W, Moverley J, Gason ASH, Heislers S (2007) Depth, sediment type, biogeography and high species richness in shallow-water benthos. Mar Freshw Res 58:293-305.

S3. Oke PR, Schiller A, Griffin DA, Brassington GB (2005) Ensemble data assimilation for an eddy-resolving ocean model. Q J Roy Meteor Soc 131: 3301-3311.

S4. Fulton EA, Smith ADM, Smith DC (2007) Alternative Management Strategies for Southeast Australian Commonwealth Fisheries: Stage 2: Quantitative Management Strategy Evaluation. Australian Fisheries Management Authority Report.

S5. van Putten IE, Gorton RJ, Fulton EA, Thebaud O (in press) The role of behavioural flexibility in a whole of ecosystem model. ICES J Mar Sci 70: 150–163

S6. Smith ADM, Sachse M, Smith DC, Prince JD, Knuckey IA, *et al* (2004) Alternative management strategies for the Southern and Eastern Scalefish and Shark Fishery – qualitative assessment report. Australian Fisheries Management Authority, Canberra.

S7. Thébaud O, Daurès F, Guyader O, Travers M, Van Iseghem S (2006) Modelling the adjustment of fishing fleets to regulatory controls: the case of South-Brittany trawlers (France), 1990-2003. GDR AMURE, Document de travail D13-2006, Brest. 13pp.

S8. Newell RG, Sanchirico JN, Kerr S (2005) Fishing quota markets. J Environ Econ Manage 49:437-462.

S9. Cope JM, Piner K, Minte-Vera CV, Punt AE (2004) Status and Future Prospects for the Cabezon (*Scorpaenichthys marmoratus*) as Assessed in 2003. Pacific Fishery Management Council, Portland. 127pp.

S10. Smith ADM, Smith DC, Tuck GN, Klaer N, Punt AE *et al* (2008) Experience in implementing harvest strategies in Australia's south-eastern fisheries. Fish Res 94: 373-379.

S11. Pantus F (2007) Sensitivity Analysis for Complex Ecosystem Models*.* PhD Thesis, School of Physical Sciences. University of Queensland.

S12. Kramer-Schadt S, Revilla E, Wiegand T, Grimm V (2007) Patterns for parameters in simulation models. Ecological Modelling 204: 553-556.

S13. Dambacher J, Luh HK, Li H, Rossignol P (2003) Qualitative stability and ambiguity in model ecosystems. Am Nat 161: 876-888.

S14. Luczkovich JL, Borgatti SP, Johnson JJ, Everett MG (2003) Defining and measuring trophic role similarity in food webs using regular equivalence. J theor Biol 220: 303–321.**Supporting Information Figure Captions**

Figure S1: Spatial management maps for bottom contact fisheries in the different management strategies: (a) status quo, (b) enhanced quota, (c) integrated and (d) conservation dominated. Key indicates percentage of the box open to fishing. Bold line indicates the boundary of main longline fisheries. Minor fisheries could have further restrictions, whereas surface and midwater fisheries typically had fewer restrictions and could access much of the area.

Figure S2: Comparison of catch per unit effort time series for Atlantis-SE versus actual historic time series for blue warehou.

Figure S3: Comparison of longline effort time series for Atlantis-SE versus actual historic time series.

Figure S4: Annual effort per depth stratum per gear type per management strategy

Figure S5: Proportional distribution of trips per month across sectors in the final 5 years of each management strategy.

Figure S6: Proportion of boats per sector exiting the fishery under the integrated management strategy.

Figure S7: Relative final biomass for the major types of biological components under each management strategy. Dark band shows interquartile range and lighter bands across all parameterisations and the lighter bands indicate the range containing > 95% of the results.

**Supporting Information Tables**

Table S1: Ecological components included in Atlantis-SE

| **Model Component** | **Group Composition** |  | **Model Component** | **Group Composition** |
| --- | --- | --- | --- | --- |
| *Pelagic invertebrates* |  |  | *Fin-fish* |  |
| Large phytoplankton | Diatoms |  | Small pelagics | *Engraulis*, *Sardinops*, sprat |
| Small phytoplankton | Picophytoplankton |  | Red bait | Emmelichthyidae |
| Small zooplankton | Heterotrophic flagellates |  | Mackerel | *Trachurus declivis*, *Scomber australisicus* |
| Mesozooplankton | Copepods |  | Migratory mesopelagics | Myctophids |
| Large zooplankton | Krill and chaetognaths |  | Non-migratory mesopelagics | Sternophychids, cyclothene (lightfish) |
| Gelatinous zooplankton | Salps (pryosomes), coelenterates |  | School whiting | *Sillago* |
| Pelagic bacteria | Pelagic attached and free-living bacteria |  | Shallow water piscivores | *Arripis*, *Thyrsites atu*, *Seriola*, leatherjackets |
| Squid | *Sepioteuthis australis*, *Notodarus gouldi* |  | Blue warehou | *Seriolella brama* |
|  |  |  | Spotted warehou | *Seriolella punctata* |
| *Benthic invertebrates* |  |  | Tuna and billfish | *Thunnus*, *Makaira*, *Tetrapturus*, *Xiphias* |
| Sediment bacteria | Aerobic and anaerobic bacteria |  | Gemfish | *Rexea solandri* |
| Carnivorous infauna | Polychaetes |  | Shallow water demersal fish | Flounder, *Pagrus auratus*, Labridae, *Chelidonichthys kumu*, *Pterygotrigla*, *Sillaginoides punctata*, *Zeus faber* |
| Deposit feeders | Holothurians, echinoderms, burrowing bivalves |  | Flathead | *Neoplatycephalus richardsoni*, *Platycephalus* |
| Deep water filter feeders | Sponges, corals, crinoids, bivalves |  | Redfish | *Centroberyx* |
| Shallow water filter feeders | Mussels, oysters, sponges, corals |  | Morwong | *Nemadactylus* |
| Scallops | *Pecten fumatus* |  | Ling | *Genypterus blacodes* |
| Herbivorous grazers | Urchins, Haliotis, gastropods |  | Blue grenadier | *Macruronus novaezelandiae* |
| Deep water megazoobenthos | Crustacea, asteroids, molluscs |  | Blue-eye trevalla | *Hyperoglyphe Antarctica* |
| Shallow water megazoobenthos | Stomatopods, octopus, seastar, gastropod, and non-commercial crustaceans |  | Ribaldo | *Mora moro* |
| Rock lobster | *Jasus edwardsii*, *Jasus verreauxi* |  | Orange roughy | *Hoplostethus atlanticus* |
| Meiobenthos | Meiobenthos |  | Dories and oreos | Oreosomatidae, Macrouridae, *Zenopsis* |
| Macroalgae | Kelp |  | Cardinalfish | Cardinalfish |
| Seagrass | Seagrass |  |  |  |
| Prawns | *Haliporoides sibogae* |  | *Sharks* |  |
| Giant crab | *Pseudocarcinus gigas* |  | Gummy shark | *Mustelus antarcticus* |
|  |  |  | School shark | *Galeorhinus galeus* |
| *Top predators* |  |  | Demersal sharks | *Heterodontus portusjacksoni*, Scyliorhinidae, Orectolobidae |
| Seabirds | Albatross, shearwater, gulls, terns, gannets, penguins |  | Pelagic sharks | *Prionace glauca*, *Isurus oxyrunchus*, *Carcharodon carcharias*, *Carcharhinus* |
| Seals | *Arctocephalus pusillus doriferus*, *Arctocephalus forsteri* |  | Dogfish | Squalidae |
| Sea lion | *Neophoca cinerea* |  | Gulper sharks | *Centrophorus* |
| Dolphins | Delphinidae |  | Skates and rays | Rajidae, Dasyatidae |
| Orcas | *Orcinus orca* |  |  |  |
| Baleen whales | *Megaptera novaeangliae*, *Balaenoptera*, *Eubalaena australis* |  |  |  |
|  |  |  |  |  |

Table S2: Fisheries (fleets and fleet components) represented in Atlantis-SE - recreational fishing includes fishing from charter boats. Each fleet can also have sub-fleets (defined on vessel size) that can act independently. Depths represents potential depths fished, fisheries did not automatically fish all potential depths at any one time or even during the course of an entire run. Note that fisheries could target many more groups than just the primary target and that the primary target group is for the start of the dynamic runs, within a run the identity of the primary target group could change as a result of decisions made by the dynamic fisheries.

| **Fishery (fleet)** | **Fleet Component** | **Gear** | **Depths (m)** | **Primary target group(s)** |
| --- | --- | --- | --- | --- |
| Dive |  | Dive | < 35 | Grazers, lobster, deposit feeders |
| Fin-fish auto-longline |  | Auto-longline | 150 - 600 ^a^ | Ling, blue grenadier, blue-eye trevalla |
| Fin-fish drop line |  | Drop lines | 150 - 650 | Blue-eye trevalla |
| Fin-fish mesh net |  | Mesh nets | 150 - 250 | Warehou |
| Fin-fish trap |  | Traps | 150 - 550 | Ling and demersals |
| Inshore line |  | Drop and hand lines | < 200 | Shallow piscivores |
| Pots |  | Traps | < 250 | Lobster, shallow megazoobenthos |
| Recreational  (represented as a tithe) |  | Multiple | < 200 | Multiple |
| Scallop dredge |  | Dredge | < 150 ^b^ | Scallops |
| Shark net |  | Mesh nets | < 150 ^c^ | Gummy shark, school shark |
| Shark longline |  | Longline | < 150 ^c^ | Gummy shark, school shark |
| Small pelagic state fisheries |  | Net, seine | < 250 | Small pelagics, mackerel |
| Small pelagic commonwealth fishery |  | Midwater trawl | < 300 | Mackerel, red bait |
| Small pelagic purse seine |  | Purse seine | < 250 | Small pelagics, mackerel |
| Squid jig |  | Jig | < 200 | Squid |
| Tuna longline |  | Pelagic longline | > 50 | Tuna and billfish |
| Tuna purse seine |  | Purse seine | > 50 | Tuna and billfish |
| Trawl (with state, SET and | Cephalopod trawl | Bottom trawl | < 300 | Squid |
| GABT sub- divisions) | Crustacean trawl | Bottom trawl | 50 – 250 | Crustaceans ^E^ |
|  | Prawn trawl | Bottom trawl | 300 - 500 | Royal red prawns |
|  | Fin-fish midwater trawl | Midwater trawl | 50-400 | Demersals |
|  | Squid midwater trawl | Midwater trawl | < 500 | Squid |
|  | Danish seine | Danish seine | < 200 | Flathead |
|  | General demersal (slope) trawl ^F^ | Bottom trawl | < 650 | Ling, blue grenadier |
|  | Shelf demersal trawl | Bottom trawl | < 250 | Flathead |
|  | Orange roughy trawl | Bottom trawl | < 1250 | Orange roughy |

A. In reality auto-longline is between 183-600m, but the resolution of the model meant that it had to be represented as either 150-600 or 250-600. It was decided in this case to use 150-600, but in the future sensitivity to this decision (or better still resolving the model so it can represent say 180-600) needs to be considered – see discussion of the gillnet and auto-longline and shark catch results for further exploration of this topic.

B. This depth was set to capture historical catches and because of the vertical resolution of the model, more recently the majority of observed scallop dredging is in waters <80m.

C. This depth was set to capture historical catches and because of the vertical resolution of the model, since the adoption of quota management for gummy and school shark most observed effort is in waters <80m.

D. The state fishery components were really only active for Crustacean trawl and Shelf demersal trawl components.

E. For state fisheries the primary target groups are prawns and giant crab, while for the Commonwealth fisheries the target group is “non prawn crustaceans”.

F. While active on the upper slope this trawl fleet ranges more widely and can be found fishing the shelf break and on the shelf (changing its targeting appropriately).
